# Novel fluorinated carbonic anhydrase IX inhibitors reduce hypoxia-induced acidification and clonogenic survival of cancer cells

**DOI:** 10.18632/oncotarget.25508

**Published:** 2018-06-01

**Authors:** Justina Kazokaitė, Raymon Niemans, Virginija Dudutienė, Holger M. Becker, Jānis Leitāns, Asta Zubrienė, Lina Baranauskienė, Gabor Gondi, Reinhard Zeidler, Jurgita Matulienė, Kaspars Tārs, Ala Yaromina, Philippe Lambin, Ludwig J. Dubois, Daumantas Matulis

**Affiliations:** ^1^ Department of Biothermodynamics and Drug Design, Institute of Biotechnology, Vilnius University, Vilnius, Lithuania; ^2^ Department of Radiotherapy (The M-Lab Group), GROW – School for Oncology and Developmental Biology, Maastricht Comprehensive Cancer Centre, Maastricht University Medical Centre, Maastricht, The Netherlands; ^3^ Department of Physiological Chemistry, University of Veterinary Medicine Hannover, Hannover, Germany; ^4^ Latvian Biomedical Research and Study Center, Riga, Latvia; ^5^ Department of Gene Vectors, Helmholtz Center for Environmental Health, Munich, Germany; ^6^ Department of Otorhinolaryngology, Klinikum der Universität München, Munich, Germany

**Keywords:** cancer, hypoxia, drug design, sulfonamide, carbonic anhydrase IX

## Abstract

Human carbonic anhydrase (CA) IX has emerged as a promising anticancer target and a diagnostic biomarker for solid hypoxic tumors. Novel fluorinated CA IX inhibitors exhibited up to 50 pM affinity towards the recombinant human CA IX, selectivity over other CAs, and direct binding to Zn(II) in the active site of CA IX inducing novel conformational changes as determined by X-ray crystallography. Mass spectrometric gas-analysis confirmed the CA IX-based mechanism of the inhibitors in a CRISPR/Cas9-mediated CA IX knockout in HeLa cells. Hypoxia-induced extracellular acidification was significantly reduced in HeLa, H460, MDA-MB-231, and A549 cells exposed to the compounds, with the *IC*_50_ values up to 1.29 nM. A decreased clonogenic survival was observed when hypoxic H460 3D spheroids were incubated with our lead compound. These novel compounds are therefore promising agents for CA IX-specific therapy.

## INTRODUCTION

Tumor hypoxia promotes invasiveness and is associated with resistance to chemotherapeutics and radiation and thus poor prognosis [1–5]. Human carbonic anhydrase IX (CA IX) shows limited expression in normal tissues [6] and is significantly up-regulated by hypoxia-inducible factor 1α (HIF-1α) [7] or other alternative microenvironmental factors [8–12] in a variety of tumors. CA IX is crucial for cancer cell survival because bicarbonate and protons, produced upon CA IX-catalyzed reversible hydration of CO_2_, are necessary to maintain the cellular pH balance: bicarbonate is transported into the cell to neutralize intracellular acid, while protons increase extracellular acidification [13–16]. CA IX also stimulates cell spreading and epithelial-mesenchymal transition [17, 18]. Therefore, CA IX has been proposed to be a promising tumor hypoxia biomarker for diagnostic and targeted drug delivery applications [19].

**Scheme 1:**
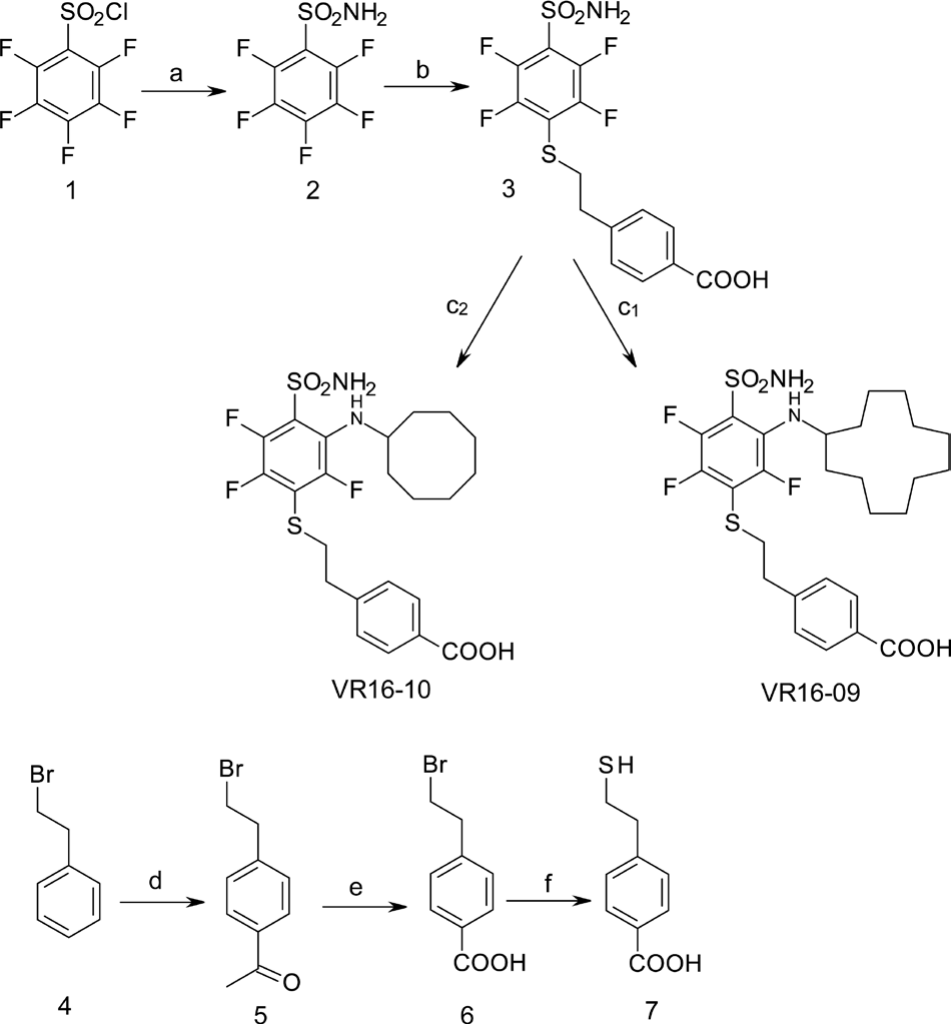
Synthesis scheme of VR16-09 and VR16-10 Reagents and conditions: (a) NH_3_ aq, THF, –10° C; (b) 7, Et_3_N, MeOH, r.t. 24 h, then conc. HCl; (c_1_) cyclododecylamine, Et_3_N, DMSO, 70° C, 36 h, then H_2_O, HCl; (c_2_) cyclooctylamine, Et_3_N, DMSO, 70° C, 24 h, then H_2_O, HCl; (d) CH_3_COCl, AlCl_3_, CH_2_Cl_2_, 0° C – +2° C, then conc. HCl; (e) NaOH, H_2_O, dioxane, Br_2_, 0° C – +2° C, then conc. HCl, (f) NH_2_CSNH_2_, H_2_O, reflux 3 h, then NaOH 1 h reflux, then 2M HCl.

Sulfonamides are classical CA inhibitors, where the deprotonated sulfonamide group is required for displacement of the catalytic Zn^2+^-bound water molecule to bind directly with Zn^2+^ in the active site to inhibit CA [20, 21]. Therefore, the binding affinity can be enhanced using inhibitors with the lowered p*K_a_* of the sulfonamide group [21, 22]. Introduction of fluorine atoms that lower the p*K_a_* due to their electron-withdrawing capabilities is one of the choices due to unique features, such as high electronegativity, small size, low atomic weight, and contribution to increased lipophilicity. Fluorine is found in ~20% of current pharmaceuticals and this trend is increasing [23]. Krishnamurthy and colleagues investigated fluorinated benzensulfonamides and concluded that fluorine is the best choice for electron-withdrawing substituents [21]. We expanded this strategy and created new routes for functionalization of pentafluorobenzensulfonamides [24, 25] including *para*-, *ortho*-, and *meta*- substituted fluorinated benzensulfonamides. The bulky hydrophobic groups at *ortho* or *meta* positions are necessary for the favorable hydrophobic contacts with the amino acids of CA IX binding pocket [26]. Here we present novel the *ortho*-substituted fluorinated benzenesulfonamides VR16-09 and VR16-10 (Table 1, Scheme 1) in combination with the previously chemically and biophysically characterized *meta* and *ortho*-substituted fluorinated inhibitors VD11-4-2 and VD12-09 [26]. We hypothesized that these benzenesulfonamides will exhibit high affinity and strong selectivity towards recombinant CA IX and will possess significant functional effects in cancer cell lines on reducing hypoxia-induced acidosis as well as hypoxia-dependent clonogenic survival, providing efficacious opportunity to target CA IX-expressing cells.

**Table 1:** The dissociation constants (*K*_*d*_s) of VR16-09, VR16-10, VD11-4-2, and VD12-09 for 12 recombinant catalytic domains of human active CA isoforms as determined by FTSA at pH 7.0 (37° C)

	*K_d_* (*K_i_*), nM			
CA isoform	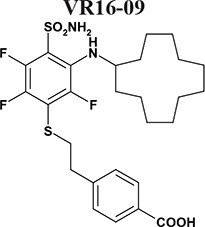	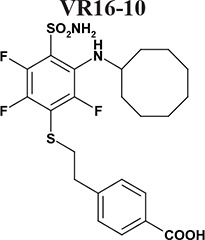	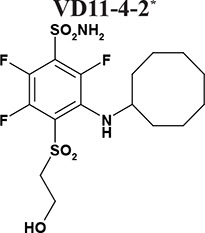	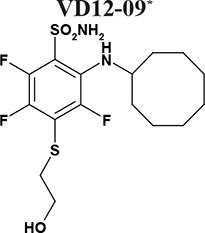
CA I	≥200 000	5000	710	50 000
CA II	≥200 000	1000	60	1 300
CA III	≥200 000	≥200 000	40 000	≥200 000
CA IV	≥200 000	1820	25	1 700
CA VA	≥200 000	5000	2 500	3 300
CA VB	45000	100	5.6	210
CA VI	≥200 000	26300	95	4 300
CA VII	37 000	100	9.8	330
CA IX	**0.16** (<1)	**0.20** (<1)	**0.05**	**1.1**
CA XII	710 (100)	370	3.3	330
CA XIII	20	40	3.6	140
CA XIV	170	170	1.6	170

The *K_i_* values determined by SFA (pH 7.5, 25^°^ C) are shown in the parentheses. Uncertainties of FTSA and SFA measurements do not exceed 2-fold.

^*^K_d_ values of VD11-4-2 and VD12-09 towards CA isoforms have been already published [26].

## RESULTS AND DISCUSSION

### Binding and inhibition of recombinant CA isoforms

**Figure 1:**
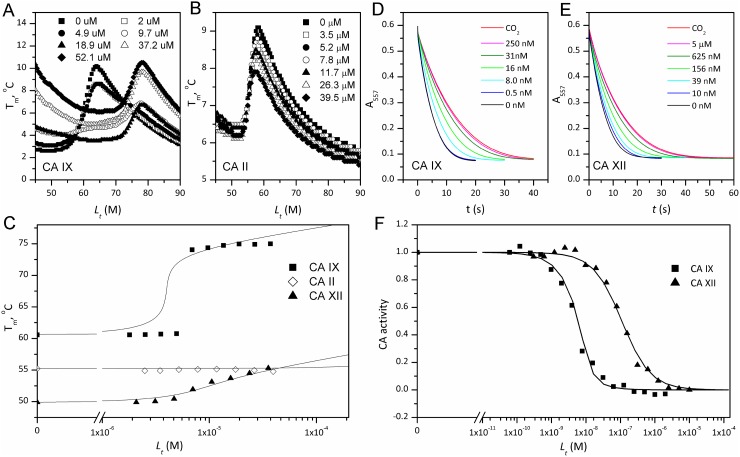
The binding affinity by FTSA (panels **A**–**C**) and inhibition by SFA (panels **D**–**F**) of VR16-09 towards human recombinant CAs. Thermal melting curves of 8 μM CA IX (A) and 5 μM CA II (B) determined by ANS fluorescence in the presence of various VR16-09 concentrations. (C) Melting temperatures (*T_m_*) of CA IX (■), CA II (◊) and CA XII (▲) as a function of added compound concentration. The lines were regressed according to [60]. Absorbance decrease due to enzymatic acidification of the medium by CA IX (D) or CA XII (E) for various VR16-09 concentrations. (F) Fraction inhibition of CA IX (■) or CA XII (▲) as a function of the total VR16-09 concentration. The lines are fit according to the Morrison equation [50].

The affinities of VR16-09 and VR16-10 to 12 catalytically active recombinant CA isoforms were determined by the fluorescent thermal-shift assay (FTSA) and compared with previously published [26] inhibitors VD11-4-2 and VD12-09 (Table 1). The *K_i_* values against CA IX and CA XII were also measured by the stopped-flow inhibition assay (SFA) of the CO_2_ hydration CA enzymatic activity (Table 1). FTSA revealed that VR16-09 bound CA IX significantly (*K_d_* = 0.16 nM) stronger than other CA isoforms (*K_d_*s > 200 μM) (Table 1, Figure 1A–1C). VR16-09 with the bulky aminocyclododecyl group exhibited greater selectivity towards CA IX as compared with VR16-10, VD12-09, and VD11-4-2 bearing aminocyclooctyl groups. SFA did not allow the determination of *K_i_* < ~2 nM against CA IX, because the concentration of CA IX was 10 nM, thus limiting the determination of *IC_50_* at 5 nM. Therefore, the *K_d_*s determined by the FTSA should be used rather than SFA. Nevertheless, SFA confirmed that VR16-09 and VR16-10 efficiently inhibited the CA IX activity (Figure 1D–1F). These compounds are advantageous compared to SLC-0111 that has entered the clinical trials (*K_i(CA IX)_* = 45 nM, *K_i(CA XII)_* = 4.5 nM and only 20-fold selectivity over CA II, thus would possibly exhibit larger adverse effects [27, 28]). In contrast, selectivity of VR16-09 for CA IX over CA I and CA II is more than one million-fold.

### Crystal structures of inhibitors bound to recombinant CA IX

**Figure 2:**
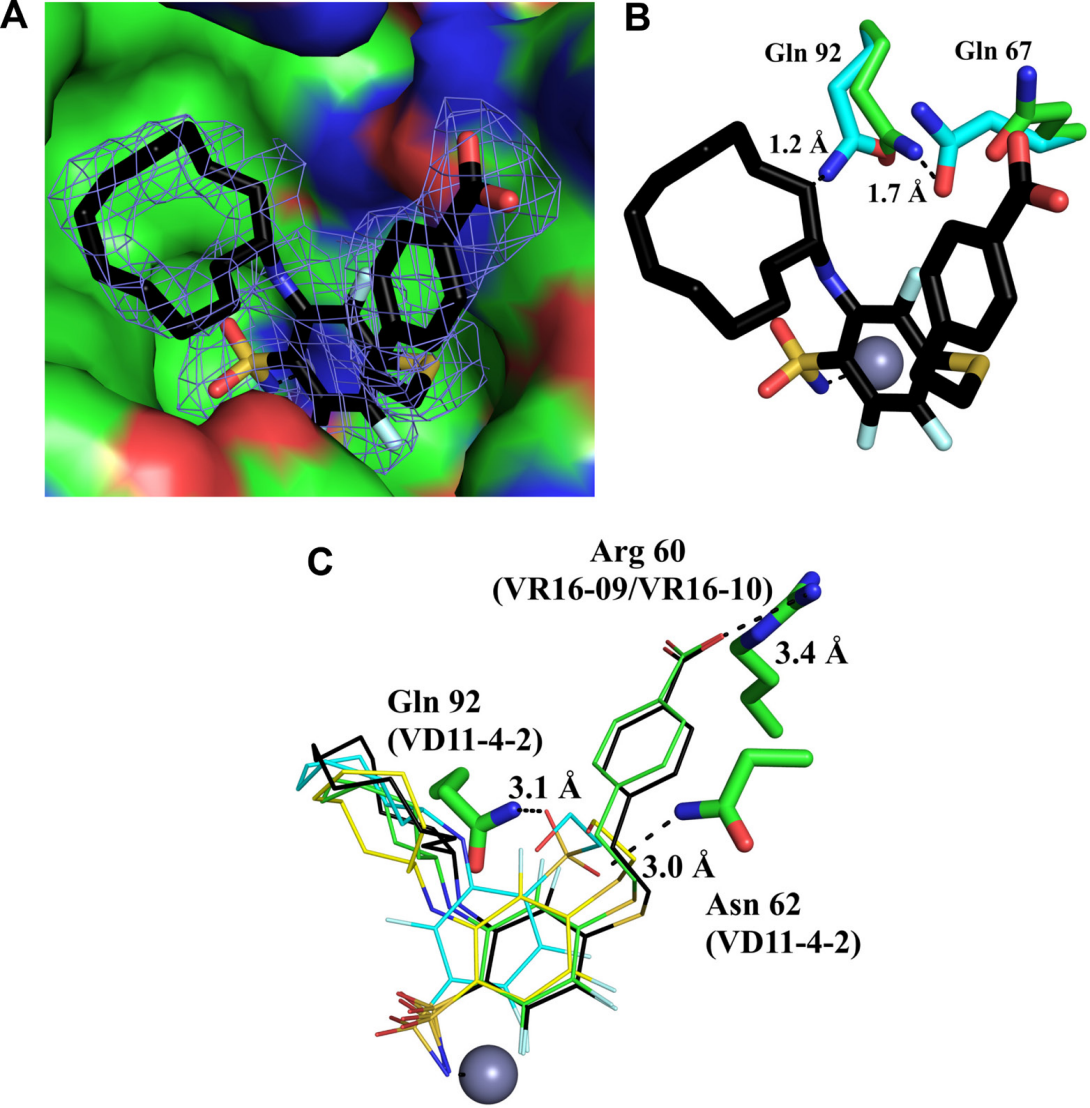
Crystal structure of VR16-09 (panel **A**, PDB ID: 6G98) bound in the active site of human recombinant CA IX. The F_o_ − F_c_ omit map is contoured at 3σ. (Panel **B**) shows that VR16-09 fitted in apo-CA IX structure (PDB ID: 6FE2), demonstrating collision points with residues indicating too short inter-atomic distances if conformational changes had not occurred. Protein and ligand carbons are shown in green and black, respectively, and apo-CA IX residues are shown in light blue. Atom colors are: oxygen (red), nitrogen (blue), fluorine (light blue), and sulfur (yellow). (Panel **C**) shows comparison of binding modes for compounds VR16-09 (black carbons), VR16-10 (green carbons), VD12-09 (yellow carbons), and VD11-4-2 (light blue carbons). The zinc ion is shown as a gray sphere and residues participating in hydrogen bonds or charged interactions are also specified for the respective inhibitors. The figure was prepared using Pymol [61].

The structures of the CA IX catalytic domain in complex with VR16-09 (PDB ID: 6G98), VR16-10 (PDB ID: 6G9U), VD12-09 (PDB ID: 6FE0) and VD11-4-2 (PDB ID: 6FE1) were determined by X-ray crystallography at resolutions ranging from 1.75 Å to 2.47 Å (Figure 2, Supplementary Table 1). The sulfonamide moiety and trifluorobenzene cycle of all observed ligands fit in the conserved region of the CA IX active site, the cycloalkane tail moieties were guided towards the hydrophobic part of the active site and moieties with terminal hydroxyl group were located in the hydrophilic part of the active site. The sulfonamide amino group formed a coordination bond with Zn(II), as observed in many other CA-sulfonamide complexes. All ligands were positioned very similarly within the active site of CA IX, except with some differences occurring in the case of VD11-4-2 (Figure 2). VD11-4-2 also formed two additional hydrogen bonds with Asn62 and Gln92, which might explain its stronger affinity for CA IX as compared to other analyzed compounds. All four crystal structures showed that some conformational changes have occurred in the CA IX active site pocket as compared to other known CA IX structures [29, 30]. In order to fully understand conformational changes occurring in those cases, we also determined apo (ligand free) CA IX structure at 1.87 Å resolution (PDB ID: 6FE2). In case of VR16-09, the cyclododecyl moiety altered rotamer of Gln92, which in turn changed the conformation of Gln67 (Figure 2).

### CA IX-dependent functional activities of inhibitors in cancer cells

**Figure 3:**
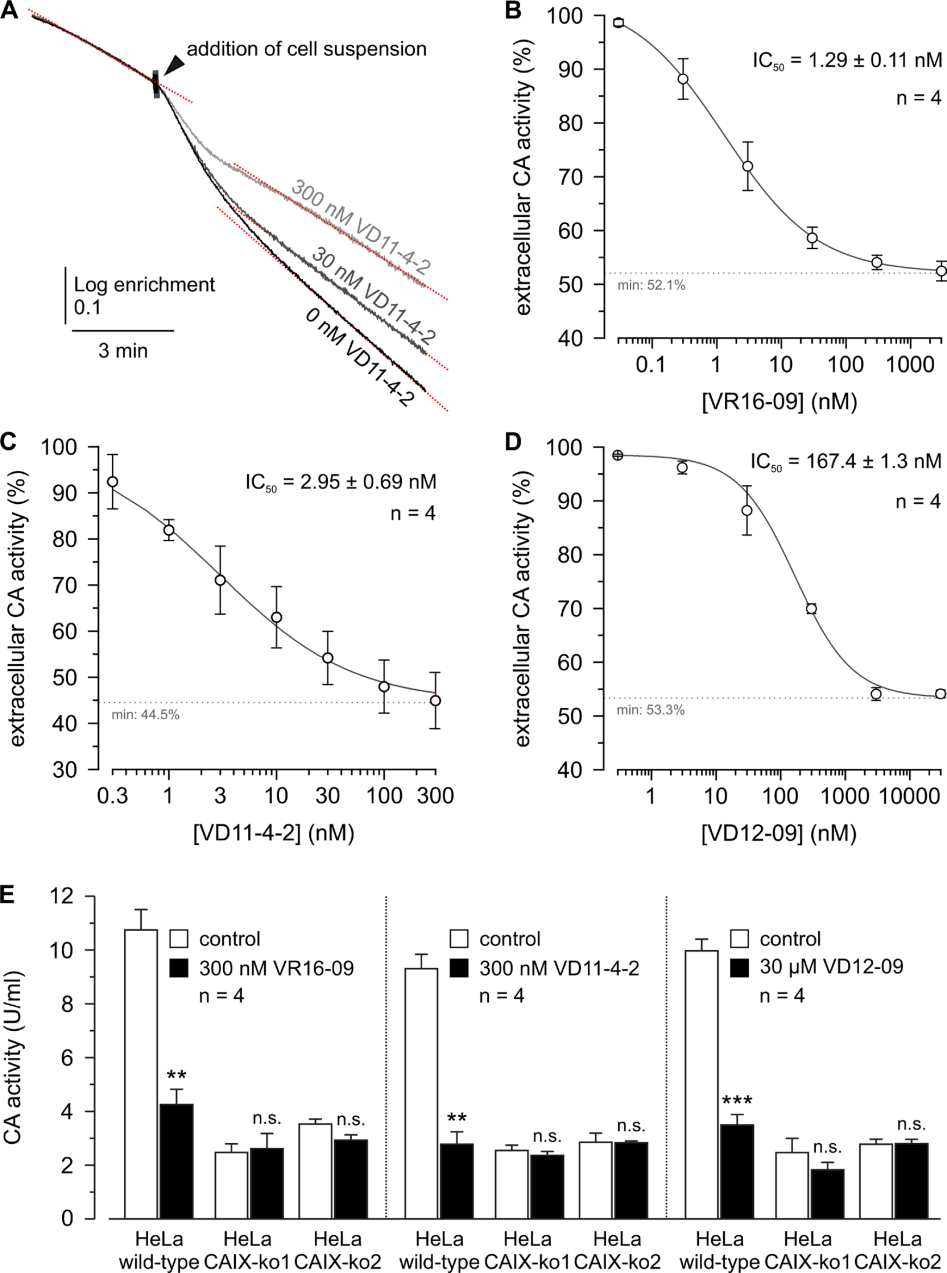
*IC_50_* determination of VR16-09, VD11-4-2, and VD12-09 in hypoxic MDA-MB-231 cells and CA IX-dependent mode of action for inhibitors in hypoxic HeLa CA IX KO cells (**A**) Original recordings of the log enrichment of MDA-MB-231 cells pre-incubated with VD11-4-2 for up to 3 h. The beginning of each trace shows the rate of degradation of the ^18^O-labeled substrate in the non-catalyzed reaction. (**B**–**D**) – Relative CA activity in MDA-MB-231 cells, incubated under hypoxia (1% O_2_) for 3 days. Cells were pre-incubated with VR16-09 (B), VD11-4-2 (C), or VD12-09 (D) for up to 3 h. CA activity was determined by MS gas-analysis from the increase in the rate of log enrichment after addition of cell suspension. CA activity in the presence of inhibitor was normalized to the activity in the absence of inhibitor. (**E**) CA activity in HeLa-WT and HeLa-CA IX-KO cells, before (white bars) and after addition of VR16-09, VD11-4-2 or VD12-09 (black bars). Average + SD of 4 independent experiments per cell line are shown. Asterisks indicate significant difference between CA activity before and after addition of inhibitor (^**^p < 0.01, ^***^p < 0.001, n.s.: not significant).

Compounds were evaluated for their biological functional activities in a panel of human cancer cell lines. CA IX expression was increased in hypoxic (0.2% O_2_) A549 (lung), AsPC-1 (pancreatic), MDA-MB-231 (breast), H460 (lung), and HeLa (cervical) cancer cells, whereas CA XII expression was similar under normoxia and hypoxia (Supplementary Figures 1, 2). We evaluated the potency of the compounds to inhibit the CA catalytic activity in hypoxic MDA-MB-231 cells by determining the rate of the CO_2_/HCO_3_^-^ hydration/dehydration reaction via ^18^O depletion from ^13^C^18^O_2_, measured by mass-spectrometric (MS) gas analysis. Addition of cell suspension resulted in an acceleration of the reaction, indicating CA catalytic activity in MDA-MB-231 cells (Figure 3A). Pre-incubation of the cell suspension with VR16-09, VD11-4-2, or VD12-09 resulted in a dose-dependent decrease in CA activity (Figure 3B–3D). To verify the CA IX specificity, hypoxic HeLa cells, in which CA IX was knocked out (KO) (Supplementary Figure 3), were exposed to VR16-09, VD11-4-2 and VD12-09 at concentrations of near maximum inhibition of extracellular CA activity in hypoxic MDA-MB-231 cells. In both HeLa CA IX KO lines, VR16-09 (300 nM), VD11-4-2 (300 nM), or VD12-09 (30 μM) did not alter CA activity (Figure 3E), although considerable CA activity remained, indicating activity of other CA isoforms. In HeLa wild-type (WT) cells, CA activity decreased (*P <* 0.01) to 30–40%, values that did not significantly differ from the two HeLa CA IX KO cell lines. Thus, VR16-09, VD11-4-2, and VD12-09 specifically target CA IX, while other CAs remain unaffected. Previously Frost and colleagues reported a *K_i_* value of 85.3 nM against extracellular CA for the fluorescent sulfonamide Cpd 5c in intact hypoxic MDA-MB-231 cells using the same ^18^O exchange assay [31]. Extracellular CA activity of MDA-MB-231 cells was also significantly reduced by 1 μM acetazolamide [32]. The inhibitors VR16-09 and VD11-4-2 exhibited higher effect towards CA IX expressed in cellular models than previously reported compounds likely due to higher affinity.

**Figure 4:**
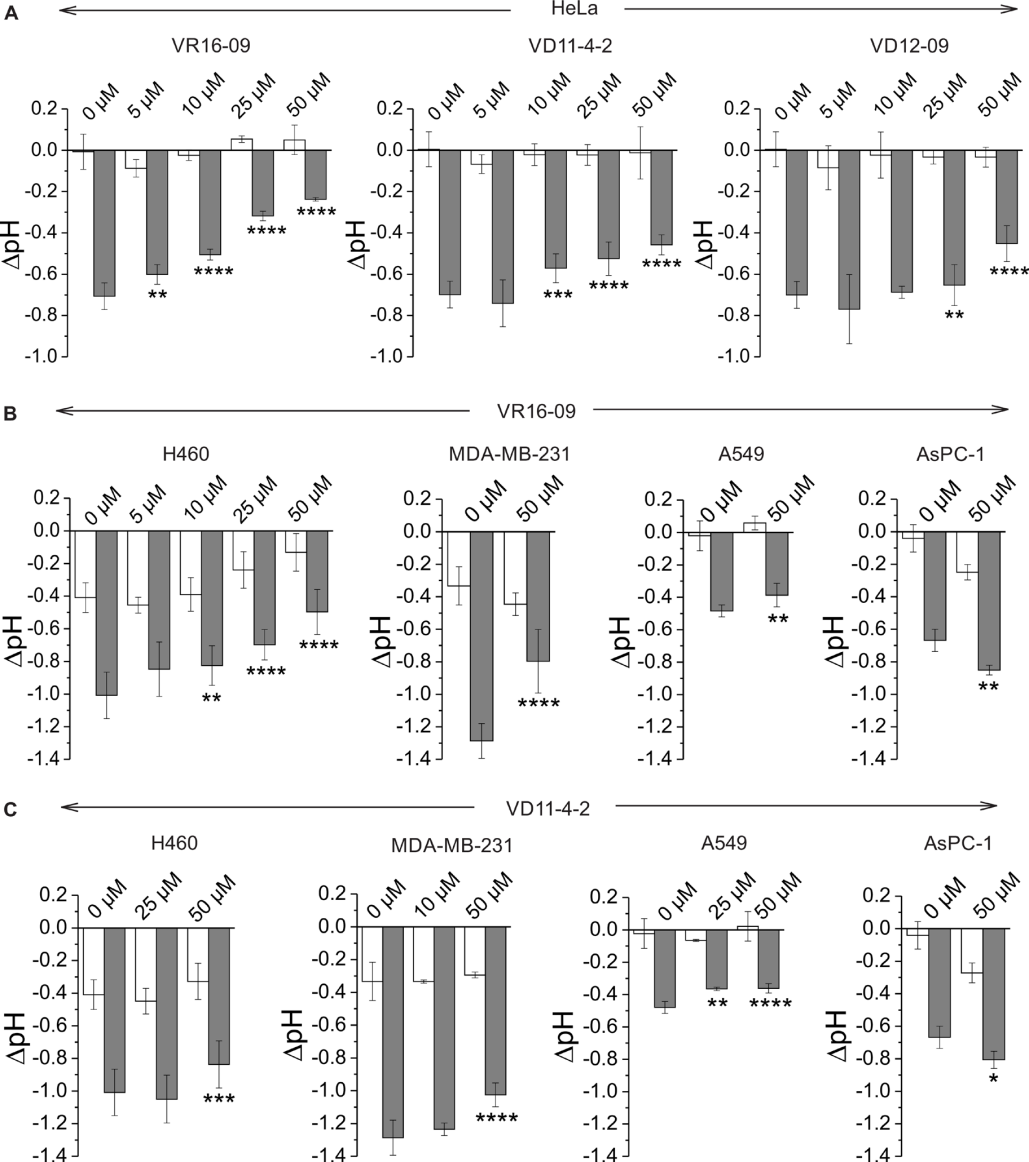
(**A**) Changes in extracellular acidification of HeLa cells after the treatment with VR16-09, VD11-4-2 or VD12-09 for 72 h under normoxia (21% O_2_; white bars) or hypoxia (0.2% O_2_; grey bars). (**B**–**C**) Effect of VR16-09 (B) and VD11-4-2 (C) on the hypoxia-induced extracellular acidification of H460, MDA-MB-231, A549, and AsPC-1 after the exposure for 72 h. Average ± SD of at least 3 independent experiments is shown. Asterisks indicate significant difference between medium pH of cells exposed to DMSO and cells treated with various doses of inhibitor under hypoxic conditions (^*^p < 0.05; ^**^p < 0.01, ^***^p < 0.001, ^****^p < 0.0001).

The functional activity of inhibitors was further confirmed by measuring the rise in extracellular pH directly inside a hypoxic chamber [33]. VR16-09, VD11-4-2, and VD12-09 significantly (*p <* 0.05) reduced hypoxia-induced acidification of HeLa cells in a dose-dependent manner, while the effect on cells exposed to normoxia was negligible (Figure 4A). This functional activity was the most pronounced for VR16-09 at 50 μM, which significantly reduced hypoxia-induced acidosis of 4 investigated cell lines (Figure 4A, 4B). VD11-4-2 also significantly reduced (*p <* 0.05) hypoxic acidification of HeLa, H460, MDA-MB-231, and A549 cells (Figure 4A, 4C). A 4-fold lower concentration of sodium bicarbonate in the medium was used for A549 to determine the functional effects of the compounds due to relatively low levels of hypoxia-induced CA IX (Supplementary Figure 1). VD12-09 exhibited functional activity in HeLa cells (Figure 4A). The smallest, albeit significant (*p <* 0.05), impact on extracellular acidification was found for VR16-10 (Supplementary Figure 4). Interestingly, inhibitors VR16-09 and VR16-10 differ structurally by the size of the hydrophobic ring at *ortho* position. Thus, a 12-carbon ring of VR16-09 was found to be more favorable than an 8-carbon ring of VR16-10 because ~3-fold higher functional effects of VR16-09 on the reduction of hypoxia-induced acidification of H460 cells were observed, even though their affinities towards recombinant CA IX were similar (*K_d_s* of 0.16 and 0.20 nM, respectively). Functional activities of several CA IX-targeting agents, such as fluorescent sulfonamide [33] and nitroimidazole-based inhibitors [34, 35] have been previously reported using the same method in HeLa cells, where a dose-dependent reduction in hypoxia-induced extracellular acidification was observed. Results of the current study indicate that the inhibitors investigated here are more efficacious in decreasing extracellular acidification than previously described compounds because of their significant functionality at lower concentrations (5–50 μM), highlighting the potential for CA IX-targeting therapy.

Interestingly, hypoxia-induced acidosis of AsPC-1 cells increased after exposure to VR16-09 or VD11-4-2 (Figure 4B, 4C), related to the unique expression of monocarboxylate transporter 1 (MCT1) under both normoxic and hypoxic conditions in these cells (Supplementary Figure 5A). Recently, CA IX was proposed to be a ‘H^+^-distributing antenna’ for MCT1 to facilitate rapid extrusion of lactate and H^+^ from the cell [36]. Even though the catalytic activity of CA IX was inhibited in AsPC-1 by compounds, the rise of acidification might be caused by MCT1 which is further non-catalytically stimulated by CA IX. In contrast, MCT4 was expressed in all tested cell lines and was up-regulated in response to hypoxia (Supplementary Figure 5B), in line with previously confirmed HIF-1α-dependent mechanism of MCT4 expression in cancer cells [37].

### Inhibitor-induced cytotoxicity

Cytotoxicity of tested compounds was higher in normoxic than hypoxic cell monolayers, as determined by cell viability assay using alamarBlue^®^ after treatment for 48 h or 72 h (Supplementary Figure 6A, 6B, Supplementary Table 2). Inhibitors were less effective in reducing viability of hypoxic CA IX-expressing cells than normoxic cells without or with significantly lower CA IX expression. Sensitivity of HeLa and H460 cells to VR16-10 was the lowest and is in line with the lowest VR16-10 functional activity measured by extracellular pH assay. Therefore, VR16-10 was not investigated further. Similarly, compounds were more effective in reducing clonogenic survival in normoxic compared to hypoxic monolayer HeLa cells (Supplementary Figure 6C). Our results correlate with previously published cytotoxicity profiles of benzenesulfonamides, including SLC-0111 [38], sulfamate S4 [39], and dual-target compounds bearing various CA IX-targeting moieties combined with different anti-cancer drugs [40], which showed more effective cell kill in normoxia than hypoxia. We hypothesize that higher cytotoxicity of inhibitors in normoxic cells as compared with hypoxic cells could be due to the affinity of investigated compounds towards CA XII, expressed in all cell lines investigated here and shown to be up-regulated to compensate the CA IX knockdown [41]. Thus, new cellular models with both CA IX and CA XII KO would be crucial to determine the link between functional activity of compounds and CA IX or CA XII-dependent cellular mechanisms.

### Hypoxia-dependent effect on spheroid clonogenic survival

**Figure 5:**
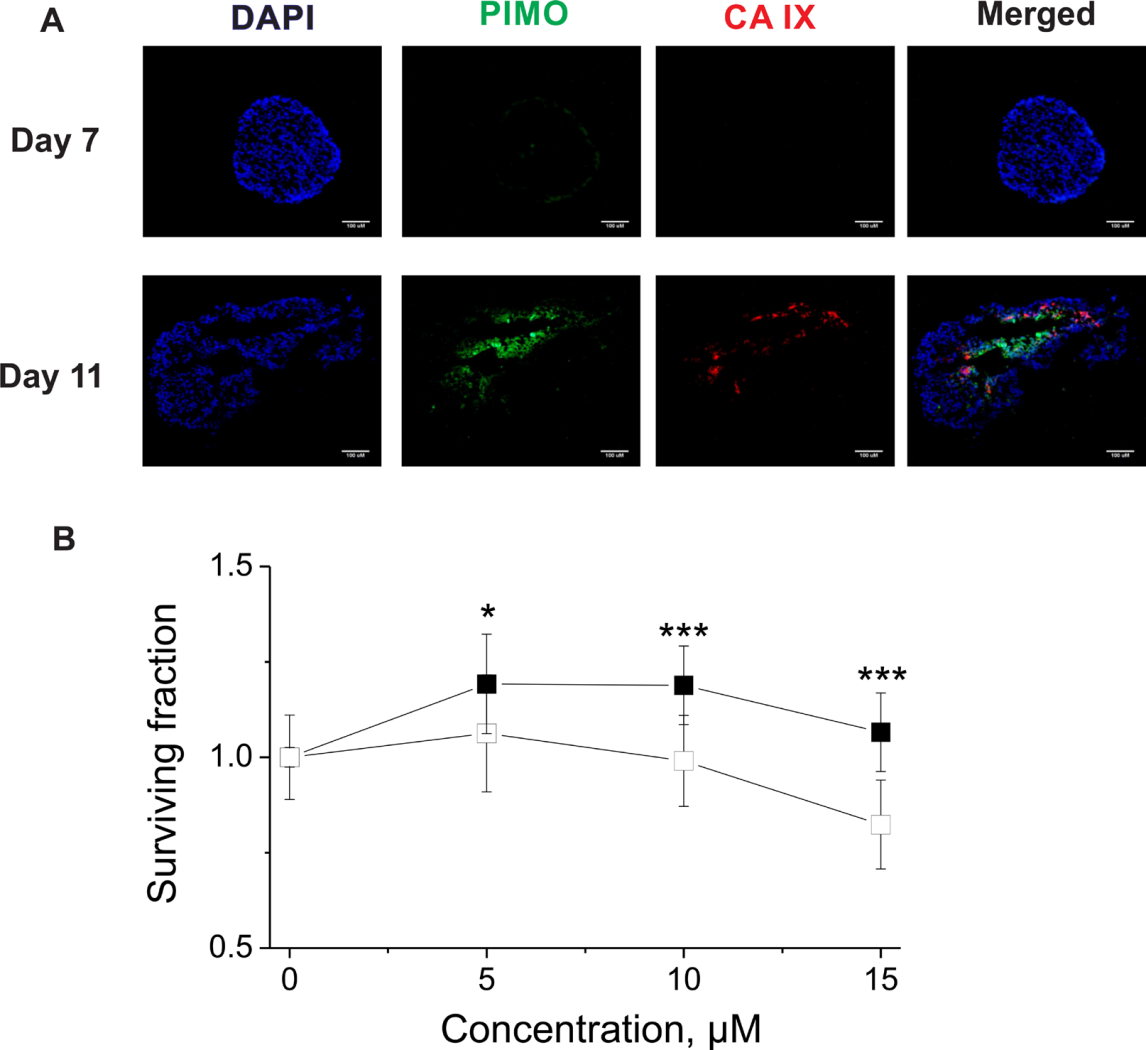
(**A**) Immunofluorescence images of H460 spheroids stained for DAPI (blue), PIMO (green) and CA IX (red). The scale bar indicates 100 μM. (**B**) Survival of clonogenic cells derived from non-hypoxic (■) and hypoxic (□) H460 spheroids exposed to VR16-09 for 24 h on day 4 or day 11, respectively. Asterisks indicate statistically significant differences between the surviving fractions of clonogenic cells derived from normoxic or hypoxic spheroids after exposure to the same dose (^*^p < 0.05, ^***^p < 0.001).

To confirm the CA IX-dependent efficacy of the compounds, H460 spheroids were employed. Immunofluorescence analysis confirmed overlap between CA IX expression and pimonidazole (PIMO)-positive hypoxia in sections of H460 spheroids grown for 11 days, whereas neither CA IX nor hypoxia were found in spheroids grown for 7 days (Figure 5A). Therefore, non-hypoxic (4 days) and hypoxic (11 days) H460 spheroids were exposed to VR16-09 using an effective dose based on extracellular pH assays (Figure 4A, 4B) for 24 h and afterwards plated for clonogenic survival. In contrast to 2D cell viability and clonogenic survival assays, a hypoxia-dependent effect on clonogenic survival of VR16-09 was found in H460 spheroids (Figure 5B). The 3D cell models reflect important properties of *in vivo* tumors such as interactions between cells, oxygen gradients, penetration of drugs, response and resistance, and production/deposition of extracellular matrix, which are absent in rapidly and uncontrollably growing 2D cells [42–44]. Our study confirms the importance of using *in vitro* 3D cellular models for screening of CA IX-targeting inhibitors.

## MATERIALS AND METHODS

### Chemistry

VR16-09 and VR16-10 were synthesized following a similar route as described in our previous research [24]. Pentafluorobenzenesulfonyl chloride (1, Acros Organics) was converted to pentafluorobenzenesulfonamide (2) via amination with aqueous ammonia (scheme 1) by our improved method [25]. On the basis of our previous investigations, sulfonamide 2 undergoes aromatic nucleophilic substitution reactions with sulfur-centered nucleophiles readily and forms exclusively the *para*-substituted products. High reactivity of polyfluorinated compounds leads to the formation of monosubstituted or even further substituted compounds. The subsequent *ortho*-substitution occurs in the case of 4-substituted-2,3,5,6-tetrafluobenzenesulfonamides, bearing non-oxidized sulfur-centered substitutes at *para* position. Similarly, sulfonamide 2 was treated with 4-(2-mercaptoethyl)benzoic acid (7) to furnish *para*-substituted compound 3 and subsequent aromatic nucleophilic substitution reactions with cyclododecylamine and cyclooctylamine in DMSO in the presence of Et_3_N yielded desirable compounds VR16-09 and VR16-10. Since suitable sulfur-centered nucleophile 7 was not commercially available, it was synthesized using literature methods. Starting from commercially available (2-bromoethyl)benzene (4, Acros Organics), Friedel-Crafts acylation with acetyl chloride proceeded in high yield. Acylation was accomplished as described in literature [45], except for changing highly toxic solvent carbon disulfide to more acceptable dichloromethane. Conversion of methyl ketone 5 into benzoic acid 6 was carried out via haloform reaction by the method of Foreman and McElvain [46]. Finally, benzoic acid 6 was treated with thiourea in refluxing water to generate intermediate salt, which was partitioned by adding sodium hydroxide solution as described by Takano [47].

### Compound characterization

All starting materials and reagents were commercial products. They were used without further purification. Melting points of the compounds were determined in open capillaries on a Thermo Scientific 9100 Series and are uncorrected. ^1^H and ^13^C NMR spectra were recorded on a Bruker spectrometer (400 and 100 MHz, respectively) in DMSO-d_6_ using residual DMSO signals (2.52 ppm and 40.21 ppm for ^1^H and ^13^C NMR spectra, respectively) as internal standard. ^19^F NMR spectra were recorded on a Bruker spectrometer (376 MHz) with CFCl_3_ as an internal standard. Proton, carbon and fluorine chemical shifts were expressed in parts per million (ppm) in the indicated solvent. Multiplicity was defined as s (singlet), d (doublet), t (triplet), q (quartet), dd (a doublet of doublets), ddd (a doublet of doublet of doublets), m (multiplet), br s (broad singlet). TLC was performed with silica gel 60 F_254_ aluminum plates (Merck) and visualized with UV light. Column chromatography was performed using silica gel 60 (0.040–0.063 mm, Merck). High-resolution mass spectra (HRMS) were recorded on a Dual-ESI Q-TOF 6520 mass spectrometer (Agilent Technologies). The purity of final compounds was verified by HPLC to be >95% using the Agilent 1290 Infinity instrument with a Poroshell 120 SB-C18 (2.1 mm × 100 mm, 2.7 μm) reversed-phase column.

### Pentafluorobenzenesulfonamide (2)

The solution of pentafluorobenzenesulfonyl chloride (1) (1.00 g, 3.75 mmol) and THF (60 mL) was cooled to ~ –10° C and aqueous ammonia (~1.20 mL, 25 %) was added dropwise while stirring until the solution was at pH ~7. After stirring for an additional 1 h, the solvent was removed under reduced pressure and the white solid was washed with cold H_2_O. Recrystallization was accomplished from H_2_O. Yield: 0.84 g (90 %), mp 156–157° C close to the value in the literature, mp 156° C [48]. ^1^H NMR (400 MHz, DMSO-*d_6_*): 8.48 (2H, s, SO_2_NH_2_). ^19^F NMR (376 MHz, DMSO-*d_6_*): –139.5 (2F, dd, *^1^J* = 19 Hz, *^2^J* = 6 Hz), –149.39 (1F, t, *J* = 22 Hz), –161.03 (2F, t, *J* = 20 Hz).

### 4-(2-{[4-(Aminosulfonyl)-2,3,5,6-tetrafluorophenyl]thio}ethyl)benzoic acid (3)


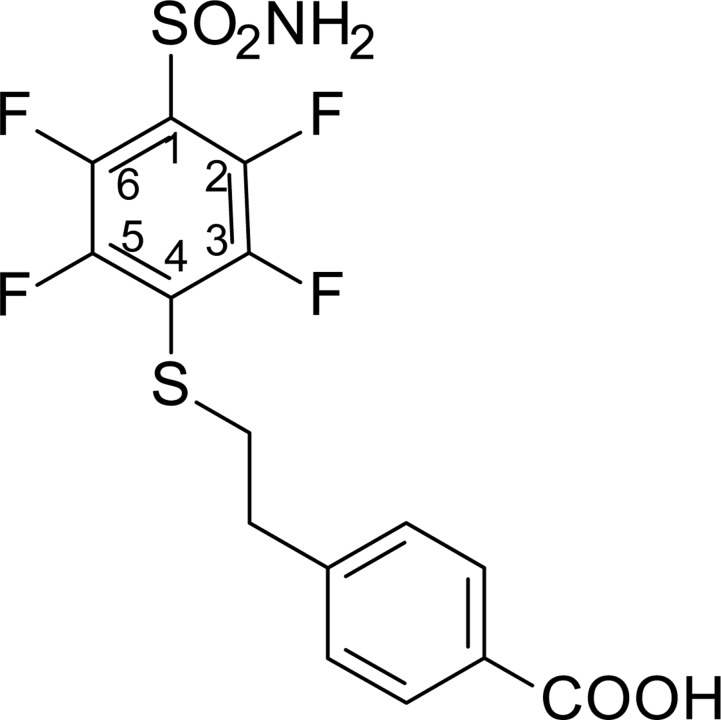


The mixture of pentafluorobenzenesulfonamide (2) (2.00 g, 8.09 mmol), 4-(2-mercaptoethyl)benzoic acid (7) (1.77 g, 9.71 mmol), Et_3_N (2.50 mL, 17.9 mmol), and MeOH (20 mL) was stirred at ambient temperature for 24 h. The solution was acidified to pH = 5 with conc. HCl and MeOH was removed under reduced pressure. The white solid was washed with water and dried. Recrystallization was accomplished from EtOH. Yield: 2.42 g (73 %), mp 235–236° C. ^1^H NMR (DMSO-*d_6_*, 400 MHz) δ 12.87 (1H, br s, COOH), 8.40 (2H, s, SO_2_NH_2_), 7.85 (2H, d, *J* = 8.1 Hz, ArH), 7.36 (2H, d, *J* = 8.1 Hz, ArH), 3.36 (2H, t, *J* = 7.3 Hz, SCH_2_CH_2_), 2.95 (2H, t, *J* = 7.3 Hz, SCH_2_CH_2_). ^13^C NMR (DMSO-*d_6_*, 100 MHz) δ 167.57 (CO), 146.85 (C3, C5, dd, *^1^J* (^19^F-^13^C) = 244.2 Hz, *^2^J* (^19^F-^13^C) = 17.1 Hz), 142.99 (C2, C6, dd, *^1^J* (^19^F-^13^C) = 254.0 Hz, *^2^J* (^19^F-^13^C) = 17.1 Hz), 144.79 (Ar), 129.78 (Ar), 129.53 (Ar), 129.28 (Ar), 122.92 (C4, t, *J* (^19^F-^13^C) = 15.3 Hz), 118.64 (C1, t, *J* (^19^F-^13^C) = 20.4 Hz), 35.91 (SCH_2_CH_2_), 34.79 (SCH_2_CH_2_). ^19^F NMR (DMSO-*d_6_*, 376 MHz) δ: –133.0 (2F, dd, *^1^J* = 24.3 Hz, *^2^J* = 9.9 Hz), –139.1 (2F, dd, *^1^J* = 24.2 Hz, *^2^J* = 9.9 Hz). HRMS for C_15_H_11_F_4_NO_4_S_2_ [(M-H)^-^]: calcd. 407.9993, found 407.9986.

### 4-(2-{[4-(Aminosulfonyl)-3-(cyclododecylamino)-2,5,6-tetrafluorophenyl]thio}ethyl)benzoic acid (VR16-09)


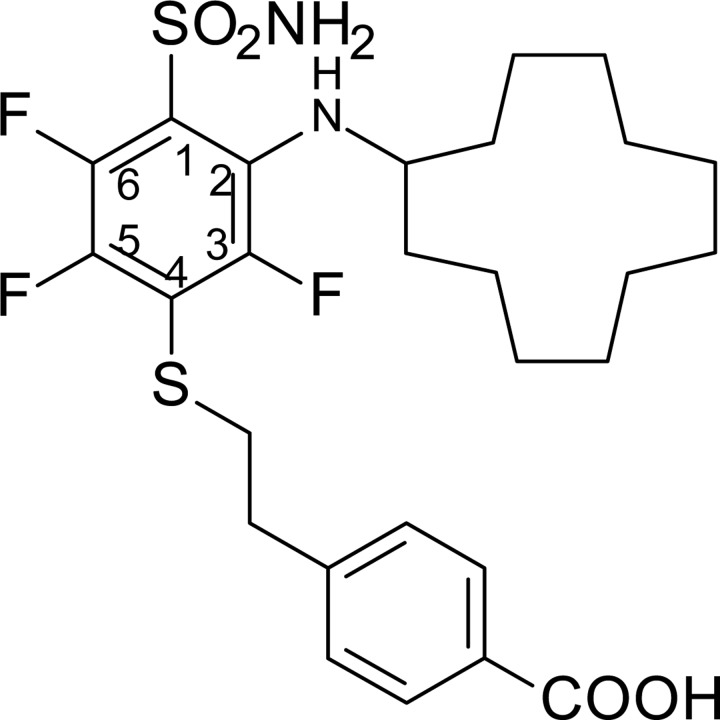


The mixture of 4-(2-{[4-(aminosulfonyl)-2,3,5,6-tetrafluorophenyl]thio}ethyl)benzoic acid (3) (0.40 g, 0.98 mmol), cyclododecylamine (0.286 g, 1.56 mmol), Et_3_N (0.34 mL, 2.44 mmol), and DMSO (6 mL) was stirred at 70° C for 36 h. The solution was cooled to room temperature, diluted with water (20 mL) and acidified to pH = 5 with 2 M HCl. The white solid was filtered, washed with water and dried. Recrystallization was accomplished from EtOH:H_2_O (2:1). Yield: 0.36 g (64 %), mp 169–170° C. ^1^H NMR (DMSO-*d_6_*, 400 MHz) δ 12.86 (1H, br s, COOH), 8.13 (2H, s, SO_2_NH_2_), 7.85 (2H, d, *J* = 8.1 Hz, ArH), 7.33 (2H, d, *J* = 8.1 Hz, ArH), 6.19 (1H, d, *J* = 9.2 Hz, NH), 3.69 (1H, br s (unresolved m), CH of cyclododecane), 3.28 (2H, t, *J* = 7.3 Hz, SCH_2_CH_2_), 2.91 (2H, t, *J* = 7.3 Hz, SCH_2_CH_2_), 1.8-1.1 (22H, m, cyclododecane). ^13^C NMR (DMSO-*d_6_*, 100 MHz) δ 167.63 (CO), 148.18 (C3, d, *J* (^19^F-^13^C) = 244 Hz), 144.84 (C6, ddd, *^1^J* (^19^F-^13^C) = 249.6 Hz, *^2^J* (^19^F-^13^C) = 15.6 Hz), *^3^J* (^19^F-^13^C) = 3.6 Hz), 144.86 (Ar), 141.62 (C5, ddd, *^1^J* (^19^F-^13^C) = 236.5 Hz, *^2^J* (^19^F-^13^C) = 15.3 Hz, *^3^J* (^19^F-^13^C) = 4.6 Hz), 132.81 (C2, d, *J* (^19^F-^13^C) = 15.1Hz), 129.78 (Ar), 129.57 (Ar), 129.14 (Ar), 119.53 (C1, dd, *^1^J* (^19^F-^13^C) = 11.9 Hz, *^2^J* (^19^F-^13^C) = 4.5 Hz), 117.52 (C4, dd, *^1^J* (^19^F-^13^C) = 23.8 Hz, *^2^J* (^19^F-^13^C) = 19.1 Hz), 52.68 (CH of cyclododecane, d, *J* = 10.6 Hz), 35.85 (SCH_2_CH_2_), 34.73 (SCH_2_CH_2_), 30.95 (cyclododecane), 24.10 (cyclododecane), 23.75 (cyclododecane), 23.40 (cyclododecane), 23.23 (cyclododecane), 21.28 (cyclo dodecane). ^19^F (DMSO-*d_6_*, 376 MHz) δ –120.6 (C3-F, d, *J* = 11.3 Hz), –137.0 (C5-F, dd, *^1^J* = 27 Hz, *^2^J* = 11.5 Hz), –145.1 (C6-F, dd, *^1^J* = 27 Hz, *^2^J* = 2.5 Hz). HRMS for C_27_H_35_F_3_N_2_O_4_S_2_ [(M-H)^-^]: calcd. 571.1918, found 571.1924.

### 4-(2-{[4-(Aminosulfonyl)-3-(cyclooctylamino)-2,5,6-tetrafluorophenyl]thio}ethyl)benzoic acid (VR16-10)


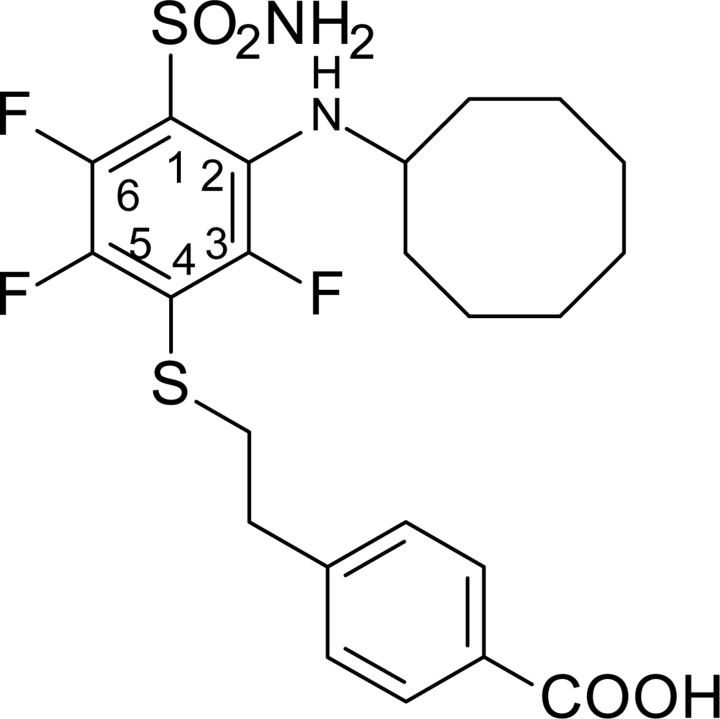


The mixture of 4-(2-{[4-(aminosulfonyl)-2,3,5,6-tetrafluorophenyl]thio}ethyl)benzoic acid (3) (0.40 g, 0.98 mmol), cyclooctylamine (0.214 mL, 1.56 mmol), Et_3_N (0.34 mL, 2.44 mmol), and DMSO (6 mL) was stirred at 70° C for 24 h. The solution was cooled to room temperature, diluted with water (20 mL) and acidified to pH = 5 with 2 M HCl. The white solid was filtered, washed with water and dried. Recrystallization was accomplished from EtOH:H_2_O (2:1). Yield: 0.32 g (64 %), mp 166–168° C. ^1^H NMR (DMSO-*d_6_*, 400 MHz) δ 12.87 (1H, br s, COOH), 8.11 (2H, s, SO_2_NH_2_), 7.85 (2H, d, *J* = 8.1 Hz, ArH), 7.33 (2H, d, *J* = 8.1 Hz, ArH), 6.33 (1H, d, *J* = 8.7 Hz, NH), 3.71 (1H, br s (unresolved m), CH of cyclooctane), 3.30 (2H, t, *J* = 7.3 Hz, SCH_2_CH_2_), 2.91 (2H, t, *J* = 7.3 Hz, SCH_2_CH_2_), 1.9-1.4 (14H, m, cyclooctane). ^13^C NMR (DMSO-*d_6_*, 100 MHz) δ 167.64 (CO), 148.16 (C3, d, *J* (^19^F-^13^C) = 242.7 Hz), 144.84 (C6, ddd, *^1^J* (^19^F-^13^C) = 249.7 Hz, *^2^J* (^19^F-^13^C) = 15.7 Hz, *^3^J* (^19^F-^13^C) = 3.8 Hz), 144.9 (Ar), 141.62 (C5, ddd, *^1^J* (^19^F-^13^C) = 236.0 Hz, *^2^J* (^19^F-^13^C) = 15.1 Hz, *^3^J* (^19^F-^13^C) = 4.4 Hz), 132.21 (C2, d, *J* (^19^F-^13^C) =14.1 Hz), 129.81 (Ar), 129.53 (Ar), 129.21 (Ar), 119.41 (C1, dd, *^1^J* (^19^F-^13^C) = 11.5 Hz, *^2^J* (^19^F-^13^C) = 4.9 Hz), 117.51 (C4, dd, *^1^J* (^19^F-^13^C) = 23.7 Hz, *^2^J* (^19^F-^13^C) = 18.5 Hz), 55.66 (CH of cyclooctane, d, *J* = 11.4 Hz), 35.86 (SCH_2_CH_2_), 34.63 (SCH_2_CH_2_), 32.62 (cyclooctane), 27.29 (cyclooctane), 25.94 (cyclooctane), 25.53 (cyclooctane), 23.38 (cyclooctane). ^19^F NMR (DMSO-*d_6_*, 376MHz) δ –120.81 (C3-F, dd, *^1^J* = 11.4 Hz, *^2^J* = 3.1 Hz), –137.1 (C5-F, dd, *^1^J* = 27.0 Hz, *^2^J* = 11.5 Hz), –145.2 (C6-F, dd, *^1^J* = 26.9 Hz, *^2^J* = 3.0 Hz). HRMS for C_23_H_27_F_3_N_2_O_4_S_2_ [(M-H)^-^]: calcd. 515.1292, found 515.1284.

### 1-[4-(2-Bromoethyl)phenyl]ethanone (5)

A mixture of AlCl_3_ (2.91 g, 21.82 mmol), acetyl chloride (1.71 mL, 24.05 mmol) and CH_2_Cl_2_ (50 mL) was stirred at 0° C – +5° C until AlCl_3_ dissolved. Solution of (2-bromoethyl)benzene (4) (2.00 mL, 14.64 mmol) in acetyl chloride (3.4 mL) was then added dropwise at 0° C – +2° C. The resulting mixture was stirred at 0° C – +2° C for 1h and then poured into a mixture of concentrated HCl (20 mL) and ice (200 mL). This mixture was extracted with CH_2_Cl_2_ (30 mL × 3). The combined organic extracts were washed with 1N NaOH, water and brine, dried over Na_2_SO_4_ and concentrated under reduced pressure. The resultant residue was subjected to flash chromatography (silica gel, hexane/EtOAc, 9:1) to afford compound 5 (2.83 g, 85 %) as an oil. ^1^H NMR (400 MHz, DMSO*-d_6_*) δ 7.94 (2H, d, *J* = 8.2 Hz, ArH), 7.33 (2H, d, *J* = 8.2 Hz, ArH), 3.61 (2H, t, *J* = 7.3 Hz, CH_2_), 3.24 (2H, t, *J* = 7.3 Hz, CH_2_), 2.60 (3H, s, COCH_3_).

### 4-(2-Bromoethyl)benzoic acid (6)

A solution of sodium hydroxide (4.16 g, 104 mmol) in H_2_O/dioxane (50 mL/40 mL) was prepared in three-necked, round bottomed flask fitted with a thermometer and a dropping funnel. The solution was cooled to 0° C in an ice-salt bath, and bromine (2.04 mL, 39.6 mmol) was slowly added with stirring at 0° C – +7° C. 1-[4-(2-Bromoethyl)phenyl]ethanone (5) (3.00 g, 13.2 mmol) was then added dropwise to sodium hypobromite solution at 0° C – +2° C. The resulting mixture was stirred at 0° C – +5° C for 1.5 h and then acidified with concentrated HCl. The precipitated acid was filtered, washed with water and dried. Recrystallization was accomplished from toluene. Yield: 2.50 g (83 %), mp 207–208° C, close to the value in the literature mp 205–207° C [46]. ^1^H NMR (400 MHz, DMSO*-d_6_*) δ 12.91 (1H, br s, COOH), 7.89 (2H, d, *J* = 8.3 Hz, ArH), 7.41 (2H, d, *J* = 8.4 Hz, ArH), 3.78 (2H, t, *J* = 7.1 Hz, CH_2_), 3.21 (2H, t, *J* = 7.1 Hz, CH_2_).

### 4-(2-Mercaptoethyl)benzoic acid (7)

The mixture of 4-(2-bromoethyl)benzoic acid (6) (2.00 g, 8.73 mmol), thiourea (0.80 g, 10.43 mmol), H_2_O (20 mL) was refluxed for 3 h. The solution was cooled to room temperature and 10 % (w/v) aqueous NaOH (18 mL) was added and the mixture was refluxed again for 1h. The solution was then cooled to room temperature, acidified to pH = 5 with 2 M HCl and filtered. The white solid was washed with water and dried. Recrystallization was accomplished from EtOH. Yield: 1.47 g (92 %), mp 157–159° C, close to the value in the literature mp 156–158° C [47]. ^1^H NMR (400 MHz, DMSO*-d_6_*) δ 12.85 (1H, br s, COOH), 7.88 (2H, d, *J* = 8.2 Hz, ArH), 7.36 (2H, d, *J* = 8.2 Hz, ArH), 2.92 (2H, t, *J* = 7.4 Hz, CH_2_), 2.77 (2H, q, *J* = 6.8 Hz, CH_2_), 2.30 (1H, t, *J* = 7.7 Hz, SH).

### CA inhibition assay

The enzymatic activity of carbonic anhydrase isoforms and their inhibition was determined by the stopped-flow CO_2_ hydration assay where the formed acid was followed by the absorbance change of a pH indicator. The Applied Photophysics SX.18MV-R stopped-flow spectrometer was used to measure the absorbance change of Phenol-Red pH indicator at 557 nm [49]. Saturated CO_2_ solution was prepared by bubbling the CO_2_ gas in MilliQ water at 25° C for 1 h. Experiments were performed at 25° C using 25 mM Hepes buffer containing 50 mM NaCl (pH 7.5), 0–10 μM compound with the final 0.4% (v/v) DMSO concentration. The final enzyme concentration was 10 nM for CA IX and 20 nM for CA XII. Raw curves of absorbance change were analyzed using Origin 8.1 (OriginLab Corporation) and the slope values were used to evaluate the rates of CO_2_ hydration. Spontaneous CO_2_ hydration rate was used as a zero value, while the CA catalyzed reaction rate - as a maximum value. The *K_d_* values were determined using the Morrison equation [50]:

$$\text{CA act}.(\%)=\left(1-\frac{([\text{CA}]+[\text{I}]+K_{d})-\sqrt{{\left([\text{CA}]+[\text{I}]+K_{d}\right)}^{2}-4[\text{CA}][\text{I}]}}{2[\text{CA}]}\right)100\%$$

### Fluorescent thermal shift assay

The binding affinity of synthesized compounds to recombinant human CAs was measured by the fluorescent thermal shift assay (FTSA) where the thermal stabilization of the protein by the compound was determined by following the fluorescence dependence on temperature at various added compound concentrations. The melting temperature (*T_m_*) of CA was measured using the Corbett Rotor-Gene 6000 instrument (QIAGEN Rotor-Gene Q, excitation at 365 ± 20 nm, emission detection at 460 ± 15 nm). The protein was heated from 25 to 99° C at a rate of 1° C/min and the fluorescence change of the solvatochromic dye 8-anilino-1-naphthalene sulfonate (ANS) was followed. The samples consisted of 5–10 μM CA, 0–200 μM ligand, 50 μM ANS, and 50 mM sodium phosphate buffer containing 100 mM NaCl at pH 7.0, with the final DMSO concentration of 2% (v/v). Protein unfolding profiles and the melting temperatures were determined at each ligand concentration while recording extrinsic fluorescence of ANS. Data analysis was performed as previously described [51]. All experiments were repeated at least twice.

### CA IX purification, crystallization and X-ray crystallography data collection

Protein was prepared and crystalized as described previously [30]. Data were collected at beamline BL14.1, Helmholtz-Zentrum Berlin, in Berlin, Germany. Images were processed by MOSFLM [52] and reflections scaled by SCALA [53]. The structure was determined by molecular replacement in MOLREP [54]. The ligand parameter files were generated by LIBCHECK [55]. Ligand modeling and manual refinement of the structure were done in COOT, followed by refinement in REFMAC. Data processing, refinement, and validation statistics are shown in Supplementary Table 1.

### Cell culture

Human cervical (HeLa), lung (H460, A549), breast (MDA-MB-231), and pancreatic (AsPC-1) cancer cells were cultured in Dulbecco's Modified Eagle's Medium (DMEM, Lonza) supplemented with 10% fetal bovine serum (FBS, Lonza). Cells were exposed to hypoxic conditions in the hypoxic chamber (MACS VA500 microaerophilic workstation, Don Whitley Scientific, UK) with 0.2% O_2_, 5% CO_2_ and residual N_2_. Simultaneously, normoxic cells were grown in the humidified incubator with 21% O_2_, 5% CO_2_ at 37° C.

### Generating HeLa CA IX knockout cells

HeLa CA IX knockout (KO) clones were established as described elsewhere [56]. HeLa cells were routinely cultured in DMEM (Lonza) supplemented with 10% FBS (Lonza) and transfected with a vector containing a CA IX-CRISPR guide RNA (CACCGGGGAATCCTCCTGCATCCG) using linear polyethylenimine (P-PEI, Polysciences Inc.). 24 h after transfection, selection with puromycin was started and maintained for 48 h, after which monoclones were picked and routinely cultured. After exposure to hypoxia (0.2%, 24 h), an initial screening for CA IX expression was performed by Western blotting (Supplementary Figure 3), followed by genetic confirmation of CA IX KO in clones that showed no CA IX expression. This was done by single allele sequencing using the TOPO^®^ TA Cloning^®^ Kit (Invitrogen) according to the manufacturer's protocol. KO-causing mutations in the *CA9* gene were confirmed in two alleles per clone.

### Determination of CA catalytic activity in cancer cells via gas-analysis mass spectrometry

Catalytic activity of CA in hypoxic MDA-MB-231 and HeLa cancer cells was determined by monitoring the ^18^O depletion of doubly labeled ^13^C^18^O_2_ through several hydration and dehydration steps of CO_2_ and HCO_3_^-^ at 24° C [57, 58]. The reaction sequence of ^18^O loss from ^13^C^18^O^18^O (m/z = 49) over the intermediate product ^13^C^18^O^16^O (m/z = 47) and the end product ^13^C^16^O^16^O (m/z = 45) was monitored with a quadrupole mass spectrometer (OmniStar GSD 320; Pfeiffer Vacuum, Asslar, Germany). The relative ^18^O enrichment was calculated from the measured 45, 47, and 49 m/z abundance as a function of time according to: log enrichment = log (49×100/(49+47+45)). For the calculation of CA activity, the rate of ^18^O depletion was obtained from the linear slope of the log enrichment over the time, using OriginPro 8.6 (OriginLab Corporation). The rate was compared with the corresponding rate of the non-catalyzed reaction. Enzyme activity in units (U) was calculated from these two values as defined by Badger and Price [59]. From this definition, one unit corresponds to 100% stimulation of the non-catalyzed ^18^O depletion of doubly labeled ^13^C^18^O_2_. MDA-MB-231 cells were cultured in Gibco Leibovitz-L15 medium (Life Technologies GmbH, Darmstadt, Germany), supplemented with 10% fetal calf serum, 5 mM glucose and 1% penicillin/streptomycin, pH 7.4. HeLa cells were cultured in RPMI-1640 Medium (Sigma Aldrich, Schnelldorf, Germany), supplemented with 10% fetal calf serum and 1% penicillin / streptomycin. Both cell lines were cultured under hypoxia (1% O_2_) for 3 days prior to measurements. Cells were trypsinized, washed and resuspended in HEPES-buffered saline (143 mM NaCl, 5 mM KCl, 2 mM CaCl_2_, 1 mM MgSO_4_, 1 mM Na_2_HPO_4_, 10 mM HEPES, pH 7.2). For determination of *IC_50_* values, 2 cell culture plates (58 cm^2^), grown to 80% confluency, were used for every single measurement. To ensure an equal amount of cells within one set of measurements, cells from several plates were pooled and then aliquoted according to the number of tested inhibitor concentrations. For the determination of *IC_50_*, the cell suspensions were incubated in the corresponding concentration of inhibitor for up to 3 h. For every measurement, the non-catalyzed reaction was determined for 6 min in the presence of inhibitor, before cell suspension was added to the measuring cuvette and the catalyzed reaction was determined for 8 min. CA activity in the presence of inhibitor was normalized to the activity in the absence of inhibitor. *IC_50_* values were determined by Hill equation using OriginPro 8.6. To investigate specificity of the inhibitors in HeLa WT and HeLa CA IX KO cells, the non-catalyzed reaction was determined for 6 min in the absence of inhibitor, before a suspension of 5 × 10^6^ cells was added to the measuring cuvette. After the catalyzed reaction was determined for 6 min, the inhibitor was added to the cuvette and the reaction was determined for another 6 min.

### Extracellular acidification (pH) assay

HeLa, H460, MDA-MB-231, and AsPC-1 cells were cultured in DMEM (Lonza) supplemented with 10% FBS (Lonza), whereas A549 cells were grown in-house made analogous medium differing only by a lower amount of sodium bicarbonate (final concentration 10 mM). Cell densities for each cell line were optimized to get ~100% confluence at the end of experiment under normoxic and hypoxic (0.2% O_2_) conditions upon vehicle (0.05% DMSO) treatment. Such conditions were necessary to obtain the highest possible level of CA IX-dependent extracellular acidification. Cells were plated in 6 cm dishes and allowed to attach overnight in normoxia. The next day cells were exposed to 5–50 μM doses of each inhibitor or DMSO for 72 h in parallel under normoxic or hypoxic conditions and pH of the culture medium was measured at the end of each experiment as previously reported [33]. Results are shown as a difference between the pH of medium in the control plate (without seeded cells) and the pH of medium in the targeted plate (cells exposed to the compound or vehicle).

### Cell viability assay

Cytotoxicity of inhibitors was determined by the alamarBlue^®^ cell viability assay (Life Technologies). Cell densities for HeLa, H460, MDA-MB-231, A549, and AsPC1 were optimized to get ~80% confluence at the end of experiment under normoxic and hypoxic (0.2% O_2_) conditions upon vehicle (0.25% DMSO) treatment. Briefly, cells were seeded in 96-well plates and allowed to attach overnight in normoxia. On the next day, cells were exposed to normoxia or hypoxia and medium was replaced with pre-incubated normoxic or hypoxic medium with final concentrations of 10–150 μM of inhibitor or DMSO. After 72 h, cells were incubated with 10% alamarBlue^®^ for 2 h under normoxia at 37° C. The fluorescence signal was measured using the multi-mode microplate reader (FLUOstar^®^ Omega, BMG Labtech) at 580 nm (excitation wavelength 540 nm). Response to treatments was quantified by evaluating *EC_50_* values (concentration of inhibitor that leads to half-maximum viability response determined by Hill fit).

### Clonogenic cell survival assays

Clonogenic survival of HeLa cell monolayers was evaluated using cell densities applied in the extracellular acidification assay to determine the effect of inhibitors on the clonogenic cell survival while having the same acidification conditions. Cells were exposed to 10–50 μM VR16-09, VD11-4-2, VD12-09, or 0.25% DMSO for 72 h upon normoxic or hypoxic conditions (0.2% O_2_). Such doses of inhibitors significantly reduced hypoxia-induced acidification. Then cells were trypsinized, reseeded in triplicate with known cell densities and allowed to form colonies for 14 days. To test inhibitors in 3D cell models, non-hypoxic and hypoxic H460 spheroids were exposed to 5–15 μM doses of VR16-09 or 0.25% DMSO for 24 h on day 4 or day 11, respectively. Single cell suspensions were prepared and cells were plated in triplicate with known cell densities and allowed to form colonies for 14 days. Colonies were quantified after staining and fixation with 0.4% methylene blue in 70% ethanol. Surviving fraction was normalized to vehicle (DMSO).

### Spheroid growth

To prepare plates for the growth of attachment-free H460 spheroids, autoclaved 1.5% w/v agarose (Sigma-Aldrich) was dispensed in the inner 60 wells of 96-well plates (50 μL/well) and left for polymerization at room temperature for 30 min. H460 cells were plated in modified 96-well plates to the surface of agarose menisci with a density of 500 cells/well. The DMEM was refreshed every two days. After 7 or 11 days in culture, spheroids were incubated with 20 μg/mL pimonidazole (PIMO, Hypoxyprobe-1, HP-1000, BioConnect) for 2 h at 37° C, collected and cryoconserved for immunofluorescence analysis (see below). In parallel 4 or 11 days after cell seeding, spheroids of homogeneous volume were treated with 5–15 μM VR16-09 or 0.25% DMSO for 24 h and collected for clonogenic survival assay.

### Western blot

Protein isolates were prepared by incubating scraped cells in RIPA buffer on ice for 30 min, followed by centrifugation to remove cell debris. Protein concentrations were determined using Bradford protein quantification reagent (BioRad). Western blot was performed using primary antibodies, including mouse anti-CA IX (M75, 1:40, kindly provided by Silvia Pastorekova, Institute of Virology, Slovak Academy of Science, Slovak Republic), mouse anti-CA XII (clone 15A4, 1:100, kindly provided by Aurelija Žvirblienė, Institute of Biotechnology, Vilnius University, Lithuania), rabbit anti-MCT1 (1:100), rabbit anti-MCT4 (1:400, kindly provided by Holger M. Becker, University of Veterinary Medicine Hannover, Hannover, Germany), rabbit anti-lamin A (1:10.000, Sigma-Aldrich), and mouse anti-actin (1:2.000.000, MP Biomedicals). Primary antibodies were incubated overnight at 4° C, whereas horseradish peroxidase-linked secondary antibodies (1:2.000, Cell Signaling) were incubated for 1 h at room temperature. Amersham ECL Western Blotting Detection Reagent (GE Healthcare Life Sciences) was applied for the detection of CA XII, MCT1, MCT4, and lamin A, while SuperSignal™ West Pico PLUS Chemiluminescent Substrate (Life Technologies) was used for the visualization of CA IX and actin.

### Immunofluorescence analysis

H460 spheroids of day 7 and day 11 were cryoconserved. Frozen sections (7 μm) of spheroids were fixed in acetone (4° C, 10 min), air-dried and rehydrated in phosphate buffered saline (PBS). Non-specific binding was blocked by incubation with 0.5% goat serum in PBS for 30 min at room temperature. Sections were stained (37° C, 1 h) using primary rabbit anti-PIMO (1:250) or mouse anti-CA IX (1:100, M75), followed by incubation (37° C, 1 h) with secondary goat anti-rabbit Alexa488 or goat anti-mouse Alexa594, respectively (both 1:500, from Invitrogen). Nuclei were stained with DAPI (final concentration 5 μg/mL) for 2 min at room temperature. Staining without primary antibody was used as negative control. Sections were viewed at 10× magnification by Nikon Eclipse E800 microscope (Nikon Instruments Inc.).

### Statistics

Statistical analysis was performed using GraphPad Prism (version 6.01). A non-parametric Mann-Whitney *U* test for small groups was applied to evaluate the statistical significance of differences between two independent groups of variables and *p <* 0.05 was assumed to be significant (^*^p < 0.05; ^**^p < 0.01, ^***^p < 0.001, ^****^p < 0.0001).

## CONCLUSIONS

In conclusion, the integrative set of synthesis, inhibitory activities, biophysical binding profiles, crystallographic analysis, and effects in 2D and 3D cancer cell culture models is described in the present study. X-ray analysis demonstrated novel, previously unseen conformational changes in CA IX active site due to ligand binding. Our compounds exhibited high affinity and selectivity towards recombinant CA IX, reached nanomolar CA IX-dependent functional effects as well reduced hypoxia-induced acidification in a variety of cancer cell lines. Interestingly, hypoxia-dependent reduction of clonogenic survival was only observed in spheroids, highlighting the importance of investigating CA IX-targeting compounds in 3D cell models resembling the naturally occurring hypoxic microenvironment with clonogenic survival as endpoint. The newly designed compounds are therefore promising agents for CA IX-specific therapy.

## SUPPLEMENTARY MATERIALS FIGURES AND TABLES



## References

[1] Harris AL (2002). Hypoxia—a key regulatory factor in tumour growth. Nat Rev Cancer.

[2] Pettersen EO, Ebbesen P, Gieling RG, Williams KJ, Dubois L, Lambin P, Ward C, Meehan J, Kunkler IH, Langdon SP, Ree AH, Flatmark K, Lyng H (2015). Targeting tumour hypoxia to prevent cancer metastasis. From biology, biosensing and technology to drug development: the METOXIA consortium. J Enzyme Inhib Med Chem.

[3] Höckel M, Vaupel P (2001). Tumor hypoxia: definitions and current clinical, biologic, and molecular aspects. J Natl Cancer Inst.

[4] Wilson WR, Hay MP (2011). Targeting hypoxia in cancer therapy. Nat Rev Cancer.

[5] Wojtkowiak JW, Verduzco D, Schramm KJ, Gillies RJ (2011). Drug resistance and cellular adaptation to tumor acidic pH microenvironment. Mol Pharm.

[6] Pastoreková S, Parkkila S, Parkkila AK, Opavský R, Zelník V, Saarnio J, Pastorek J (1997). Carbonic anhydrase IX, MN/CA IX: analysis of stomach complementary DNA sequence and expression in human and rat alimentary tracts. Gastroenterology.

[7] Wykoff CC, Beasley NJ, Watson PH, Turner KJ, Pastorek J, Sibtain A, Wilson GD, Turley H, Talks KL, Maxwell PH, Pugh CW, Ratcliffe PJ, Harris AL (2000). Hypoxia-inducible expression of tumor-associated carbonic anhydrases. Cancer Res.

[8] Simko V, Takacova M, Debreova M, Laposova K, Ondriskova-Panisova E, Pastorekova S, Csaderova L, Pastorek J (2016). Dexamethasone downregulates expression of carbonic anhydrase IX via HIF-1α and NF-κB-dependent mechanisms. Int J Oncol.

[9] Andreucci E, Peppicelli S, Carta F, Brisotto G, Biscontin E, Ruzzolini J, Bianchini F, Biagioni A, Supuran CT, Calorini L (2017). Carbonic anhydrase IX inhibition affects viability of cancer cells adapted to extracellular acidosis. J Mol Med (Berl).

[10] Ihnatko R, Kubes M, Takacova M, Sedlakova O, Sedlak J, Pastorek J, Kopacek J, Pastorekova S (2006). Extracellular acidosis elevates carbonic anhydrase IX in human glioblastoma cells via transcriptional modulation that does not depend on hypoxia. Int J Oncol.

[11] Zatovicova M, Sedlakova O, Svastova E, Ohradanova A, Ciampor F, Arribas J, Pastorek J, Pastorekova S (2005). Ectodomain shedding of the hypoxia-induced carbonic anhydrase IX is a metalloprotease-dependent process regulated by TACE/ADAM17. Br J Cancer.

[12] Závada J, Závadová Z, Zat’ovicová M, Hyrsl L, Kawaciuk I (2003). Soluble form of carbonic anhydrase IX (CA IX) in the serum and urine of renal carcinoma patients. Br J Cancer.

[13] Pastorek J, Pastoreková S, Callebaut I, Mornon JP, Zelník V, Opavský R, Zat’ovicová M, Liao S, Portetelle D, Stanbridge EJ (1994). Cloning and characterization of MN, a human tumor-associated protein with a domain homologous to carbonic anhydrase and a putative helix-loop-helix DNA binding segment. Oncogene.

[14] Svastová E, Hulíková A, Rafajová M, Zat’ovicová M, Gibadulinová A, Casini A, Cecchi A, Scozzafava A, Supuran CT, Pastorek J, Pastoreková S (2004). Hypoxia activates the capacity of tumor-associated carbonic anhydrase IX to acidify extracellular pH. FEBS Lett.

[15] Ditte P, Dequiedt F, Svastova E, Hulikova A, Ohradanova-Repic A, Zatovicova M, Csaderova L, Kopacek J, Supuran CT, Pastorekova S, Pastorek J (2011). Phosphorylation of carbonic anhydrase IX controls its ability to mediate extracellular acidification in hypoxic tumors. Cancer Res.

[16] Swietach P, Vaughan-Jones RD, Harris AL (2007). Regulation of tumor pH and the role of carbonic anhydrase 9. Cancer Metastasis Rev.

[17] Csaderova L, Debreova M, Radvak P, Stano M, Vrestiakova M, Kopacek J, Pastorekova S, Svastova E (2013). The effect of carbonic anhydrase IX on focal contacts during cell spreading and migration. Front Physiol.

[18] Svastova E, Witarski W, Csaderova L, Kosik I, Skvarkova L, Hulikova A, Zatovicova M, Barathova M, Kopacek J, Pastorek J, Pastorekova S (2012). Carbonic anhydrase IX interacts with bicarbonate transporters in lamellipodia and increases cell migration via its catalytic domain. J Biol Chem.

[19] Pastorek J, Pastorekova S (2015). Hypoxia-induced carbonic anhydrase IX as a target for cancer therapy: from biology to clinical use. Semin Cancer Biol.

[20] Gaspari R, Rechlin C, Heine A, Bottegoni G, Rocchia W, Schwarz D, Bomke J, Gerber HD, Klebe G, Cavalli A (2016). Kinetic and Structural Insights into the Mechanism of Binding of Sulfonamides to Human Carbonic Anhydrase by Computational and Experimental Studies. J Med Chem.

[21] Krishnamurthy VM, Bohall BR, Kim CY, Moustakas DT, Christianson DW, Whitesides GM (2007). Thermodynamic parameters for the association of fluorinated benzenesulfonamides with bovine carbonic anhydrase II. Chem Asian J.

[22] Krishnamurthy VM, Kaufman GK, Urbach AR, Gitlin I, Gudiksen KL, Weibel DB, Whitesides GM (2008). Carbonic anhydrase as a model for biophysical and physical-organic studies of proteins and protein-ligand binding. Chem Rev.

[23] Zhou Y, Wang J, Gu Z, Wang S, Zhu W, Aceña JL, Soloshonok VA, Izawa K, Liu H (2016). Next Generation of Fluorine-Containing Pharmaceuticals, Compounds Currently in Phase II-III Clinical Trials of Major Pharmaceutical Companies: New Structural Trends and Therapeutic Areas. Chem Rev.

[24] Dudutienė V, Zubrienė A, Smirnov A, Timm DD, Smirnovienė J, Kazokaitė J, Michailovienė V, Zakšauskas A, Manakova E, Gražulis S, Matulis D (2015). Functionalization of fluorinated benzenesulfonamides and their inhibitory properties toward carbonic anhydrases. ChemMedChem.

[25] Dudutienė V, Zubrienė A, Smirnov A, Gylytė J, Timm D, Manakova E, Gražulis S, Matulis D (2013). 4-Substituted-2,3,5,6-tetrafluorobenzenesulfonamides as inhibitors of carbonic anhydrases I, II, VII, XII, and XIII. Bioorg Med Chem.

[26] Dudutienė V, Matulienė J, Smirnov A, Timm DD, Zubrienė A, Baranauskienė L, Morkūnaite V, Smirnovienė J, Michailovienė V, Juozapaitienė V, Mickevičiūtė A, Kazokaitė J, Bakšytė S (2014). Discovery and characterization of novel selective inhibitors of carbonic anhydrase IX. J Med Chem.

[27] Lou Y, McDonald PC, Oloumi A, Chia S, Ostlund C, Ahmadi A, Kyle A, Auf dem Keller U, Leung S, Huntsman D, Clarke B, Sutherland BW, Waterhouse D (2011). Targeting tumor hypoxia: suppression of breast tumor growth and metastasis by novel carbonic anhydrase IX inhibitors. Cancer Res.

[28] Pacchiano F, Carta F, McDonald PC, Lou Y, Vullo D, Scozzafava A, Dedhar S, Supuran CT (2011). Ureido-substituted benzenesulfonamides potently inhibit carbonic anhydrase IX and show antimetastatic activity in a model of breast cancer metastasis. J Med Chem.

[29] Alterio V, Hilvo M, Di Fiore A, Supuran CT, Pan P, Parkkila S, Scaloni A, Pastorek J, Pastorekova S, Pedone C, Scozzafava A, Monti SM, De Simone G (2009). Crystal structure of the catalytic domain of the tumor-associated human carbonic anhydrase IX. Proc Natl Acad Sci USA.

[30] Leitans J, Kazaks A, Balode A, Ivanova J, Zalubovskis R, Supuran CT, Tars K (2015). Efficient Expression and Crystallization System of Cancer-Associated Carbonic Anhydrase Isoform IX. J Med Chem.

[31] Li Y, Tu C, Wang H, Silverman DN, Frost SC (2011). Catalysis and pH control by membrane-associated carbonic anhydrase IX in MDA-MB-231 breast cancer cells. J Biol Chem.

[32] Tu C, Foster L, Alvarado A, McKenna R, Silverman DN, Frost SC (2012). Role of zinc in catalytic activity of carbonic anhydrase IX. Arch Biochem Biophys.

[33] Dubois L, Douma K, Supuran CT, Chiu RK, van Zandvoort MA, Pastoreková S, Scozzafava A, Wouters BG, Lambin P (2007). Imaging the hypoxia surrogate marker CA IX requires expression and catalytic activity for binding fluorescent sulfonamide inhibitors. Radiother Oncol.

[34] Rami M, Dubois L, Parvathaneni NK, Alterio V, van Kuijk SJ, Monti SM, Lambin P, De Simone G, Supuran CT, Winum JY (2013). Hypoxia-targeting carbonic anhydrase IX inhibitors by a new series of nitroimidazole-sulfonamides/sulfamides/sulfamates. J Med Chem.

[35] Dubois L, Peeters SG, van Kuijk SJ, Yaromina A, Lieuwes NG, Saraya R, Biemans R, Rami M, Parvathaneni NK, Vullo D, Vooijs M, Supuran CT, Winum JY, Lambin P (2013). Targeting carbonic anhydrase IX by nitroimidazole based sulfamides enhances the therapeutic effect of tumor irradiation: a new concept of dual targeting drugs. Radiother Oncol.

[36] Jamali S, Klier M, Ames S, Barros LF, McKenna R, Deitmer JW, Becker HM (2015). Hypoxia-induced carbonic anhydrase IX facilitates lactate flux in human breast cancer cells by non-catalytic function. Sci Rep.

[37] Ullah MS, Davies AJ, Halestrap AP (2006). The plasma membrane lactate transporter MCT4, but not MCT1, is up-regulated by hypoxia through a HIF-1alpha-dependent mechanism. J Biol Chem.

[38] Angeli A, Tanini D, Peat TS, Di Cesare Mannelli L, Bartolucci G, Capperucci A, Ghelardini C, Supuran CT, Carta F (2017). Discovery of New Selenoureido Analogues of 4-(4-Fluorophenylureido)benzenesulfonamide as Carbonic Anhydrase Inhibitors. ACS Med Chem Lett.

[39] Meehan J, Ward C, Turnbull A, Bukowski-Wills J, Finch AJ, Jarman EJ, Xintaropoulou C, Martinez-Perez C, Gray M, Pearson M, Mullen P, Supuran CT, Carta F (2017). Inhibition of pH regulation as a therapeutic strategy in hypoxic human breast cancer cells. Oncotarget.

[40] van Kuijk SJ, Parvathaneni NK, Niemans R, van Gisbergen MW, Carta F, Vullo D, Pastorekova S, Yaromina A, Supuran CT, Dubois LJ, Winum JY, Lambin P (2017). New approach of delivering cytotoxic drugs towards CAIX expressing cells: A concept of dual-target drugs. Eur J Med Chem.

[41] Chiche J, Ilc K, Laferrière J, Trottier E, Dayan F, Mazure NM, Brahimi-Horn MC, Pouysségur J (2009). Hypoxia-inducible carbonic anhydrase IX and XII promote tumor cell growth by counteracting acidosis through the regulation of the intracellular pH. Cancer Res.

[42] Zanoni M, Piccinini F, Arienti C, Zamagni A, Santi S, Polico R, Bevilacqua A, Tesei A (2016). 3D tumor spheroid models for *in vitro* therapeutic screening: a systematic approach to enhance the biological relevance of data obtained. Sci Rep.

[43] Baker BM, Chen CS (2012). Deconstructing the third dimension: how 3D culture microenvironments alter cellular cues. J Cell Sci.

[44] Kimlin LC, Casagrande G, Virador VM (2013). *In vitro* three-dimensional (3D) models in cancer research: an update. Mol Carcinog.

[45] Wang Z, Tang J, Salomon CE, Dreis CD, Vince R (2010). Pharmacophore and structure-activity relationships of integrase inhibition within a dual inhibitor scaffold of HIV reverse transcriptase and integrase. Bioorg Med Chem.

[46] Foreman EL, McElvain SM (1940). The Reaction of Organic Halides with Piperidine. V. Negatively Substituted Ethyl Bromides. J Am Chem Soc.

[47] Takano S, Yamazoe S, Koyasu K, Tsukuda T (2015). Slow-Reduction Synthesis of a Thiolate-Protected One-Dimensional Gold Cluster Showing an Intense Near-Infrared Absorption. J Am Chem Soc.

[48] Robson P, Smith TA, Stephens R, Tatlow JC (1963). Aromatic Polyfluoro-Compounds. Part XIII. Derivatives of Penta- and 2,3,5,6-Tetra-Fluorothiophenol. J Chem Soc.

[49] Khalifah RG (1971). The carbon dioxide hydration activity of carbonic anhydrase. I. Stop-flow kinetic studies on the native human isoenzymes B and C. J Biol Chem.

[50] Smirnovienė J, Smirnovas V, Matulis D (2017). Picomolar inhibitors of carbonic anhydrase: importance of inhibition and binding assays. Anal Biochem.

[51] Baranauskienė L, Hilvo M, Matulienė J, Golovenko D, Manakova E, Dudutienė V, Michailovienė V, Torresan J, Jachno J, Parkkila S, Maresca A, Supuran CT, Gražulis S, Matulis D (2010). Inhibition and binding studies of carbonic anhydrase isozymes I, II and IX with benzimidazo[1,2-c][1,2,3]thiadiazole-7-sulphonamides. J Enzyme Inhib Med Chem.

[52] Leslie AG (1992). Recent changes to the MOSFLM package for processing film and image plate data. Newsletter on Protein Crystallography.

[53] Evans PR (1997). SCALA. Newsletter on Protein Crystallography.

[54] Vagin A, Teplyakov A (1997). MOLREP: An Automated Program for Molecular Replacement. J Appl Cryst.

[55] Vagin AA, Murshudov GN, Strokopytov BV (1998). BLANC: The Program Suite for Protein Crystallography. J Appl Cryst.

[56] Ran FA, Hsu PD, Wright J, Agarwala V, Scott DA, Zhang F (2013). Genome engineering using the CRISPR-Cas9 system. Nat Protoc.

[57] Becker HM, Hirnet D, Fecher-Trost C, Sültemeyer D, Deitmer JW (2005). Transport activity of MCT1 expressed in Xenopus oocytes is increased by interaction with carbonic anhydrase. J Biol Chem.

[58] Tu CK, Silverman DN (1982). Solvent deuterium isotope effects in the catalysis of oxygen-18 exchange by human carbonic anhydrase II. Biochemistry.

[59] Price GD, Badger MR (1989). Isolation and Characterization of High CO(2)-Requiring-Mutants of the Cyanobacterium Synechococcus PCC7942 : Two Phenotypes that Accumulate Inorganic Carbon but Are Apparently Unable to Generate CO(2) within the Carboxysome. Plant Physiol.

[60] Cimmperman P, Baranauskienė L, Jachimoviciūte S, Jachno J, Torresan J, Michailovienė V, Matulienė J, Sereikaitė J, Bumelis V, Matulis D (2008). A quantitative model of thermal stabilization and destabilization of proteins by ligands. Biophys J.

[61] (2012). The PyMOL Molecular Graphics System, version 1.5.0.1.

