# Supplementary material for: Novel fluorinated carbonic anhydrase IX inhibitors reduce hypoxia-induced acidification and clonogenic survival of cancer cells
Source: Oncotarget. 2018 Jun 1;9(42):26800–16. doi: 10.18632/oncotarget.25508 (PMC6003569; doi:10.18632/oncotarget.25508)
Supplement: Supplementary file 1 [file oncotarget-09-26800-s001.pdf]

Novel fluorinated carbonic anhydrase IX inhibitors reduce hypoxia-induced acidification and clonogenic survival of cancer cells

SUPPLEMENTARY MATERIALS

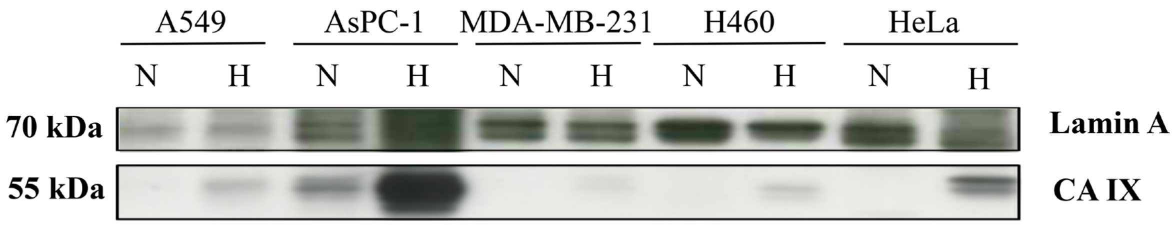

Supplementary Figure 1: Western blot analysis of CA IX expression in A549, AsPC-1, MDA-MB-231, H460, and HeLa cells after exposure to normoxia (N, 21%) or hypoxia (H, 0.2%) for 72 h. Lamin A was used as loading control.

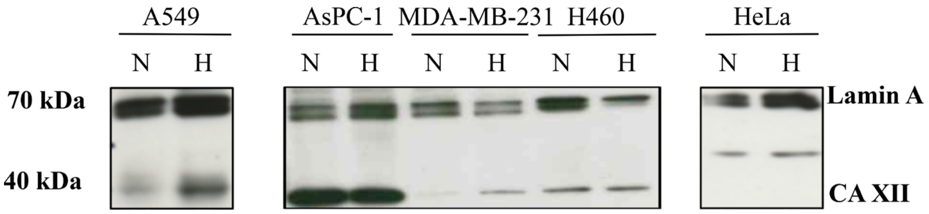

Supplementary Figure 2: Western blot analysis of CA XII expression in A549, AsPC-1, MDA-MB-231, H460, and HeLa cells after exposure to normoxia (N, 21%) or hypoxia (H, 0.2%) for 72 h. The MW of CA XII in HeLa was found to be larger than in other tested cell lines, suggesting variability of CA XII isoforms or their post-translational modifications in different cell lines. Lamin A was used as loading control.

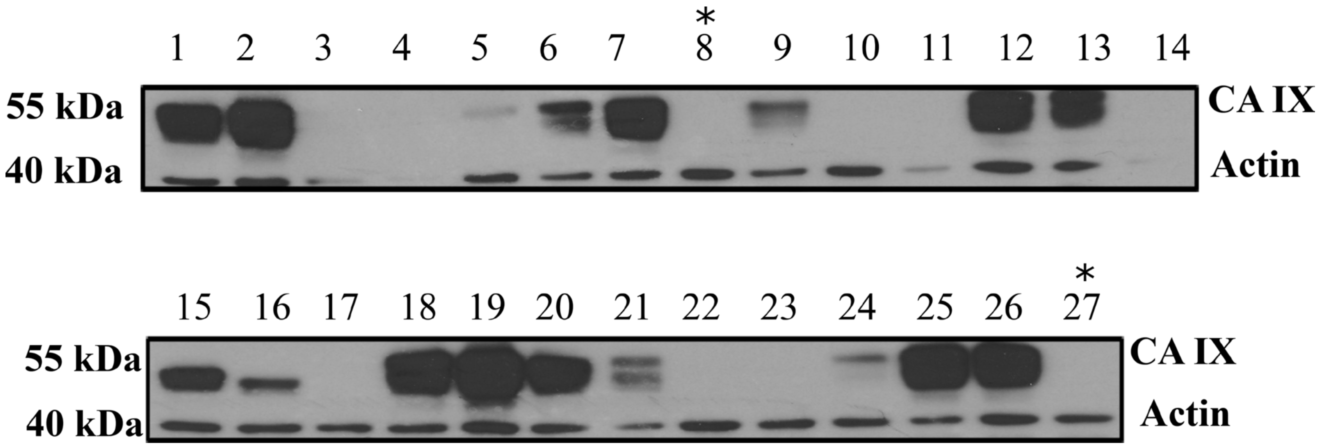

Supplementary Figure 3: Western blot analysis of CA IX expression in a panel of expected HeLa CA IX KO clones after exposure to hypoxia (0.2%) for 24 h. Clones 8 and 27, marked by asterisks, represent selected HeLa CA IX KO clones 1 and 2, respectively, which were used in experiments after genetic confirmation of KO-causing mutations in the *CA9* gene in two alleles per clone. The growth rate of clones 8 and 27 were similar as compared to HeLa WT cells. Actin was used as loading control.

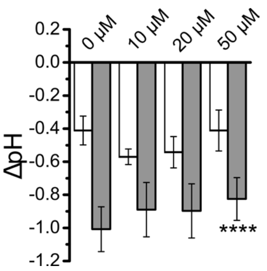

Supplementary Figure 4: Dose-dependent reduction of extracellular acidification of H460 cells after VR16-10 exposure for 72 h under normoxia (21% O_2; white bars) or hypoxia (0.2% O_2; grey bars). Results are shown as a difference between the pH of medium in the control plate (DMEM without seeded cells) and the pH of medium in the targeted plate (H460 exposed to the compound or vehicle) after the incubation for 72 h. Asterisks indicate significant difference of medium pH of cells exposed to DMSO and cells treated with various doses of inhibitor under hypoxic conditions ($^{****}p < 0.0001$).

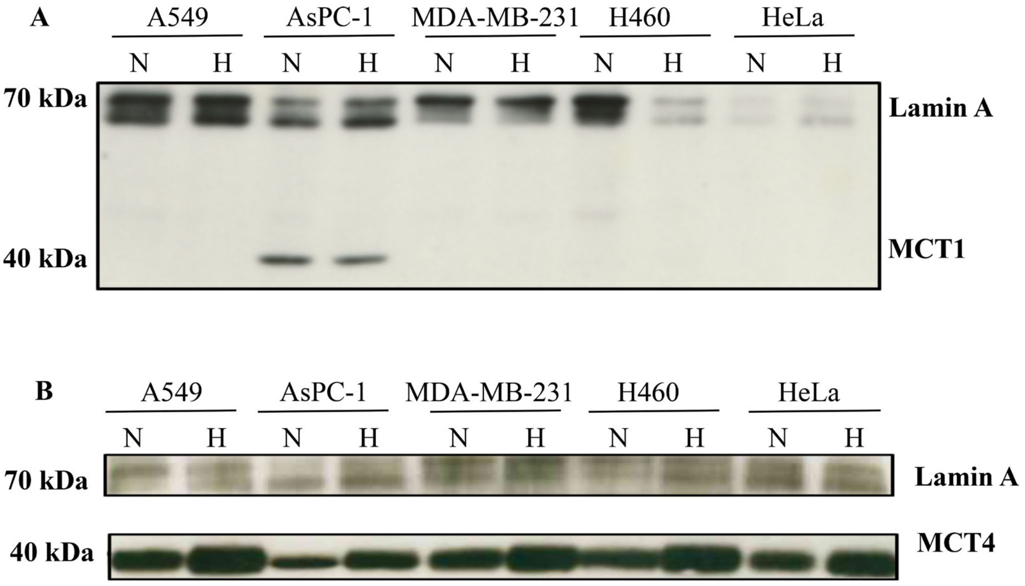

Supplementary Figure 5: Western blot analysis of MCT1 (**A**) and MCT4 (**B**) expression in A549, AsPC-1, MDA-MB-231, H460, and HeLa cell lines after exposure to normoxia (N, 21%) or hypoxia (H, 0.2%) for 72 h. Lamin A was used as loading control.

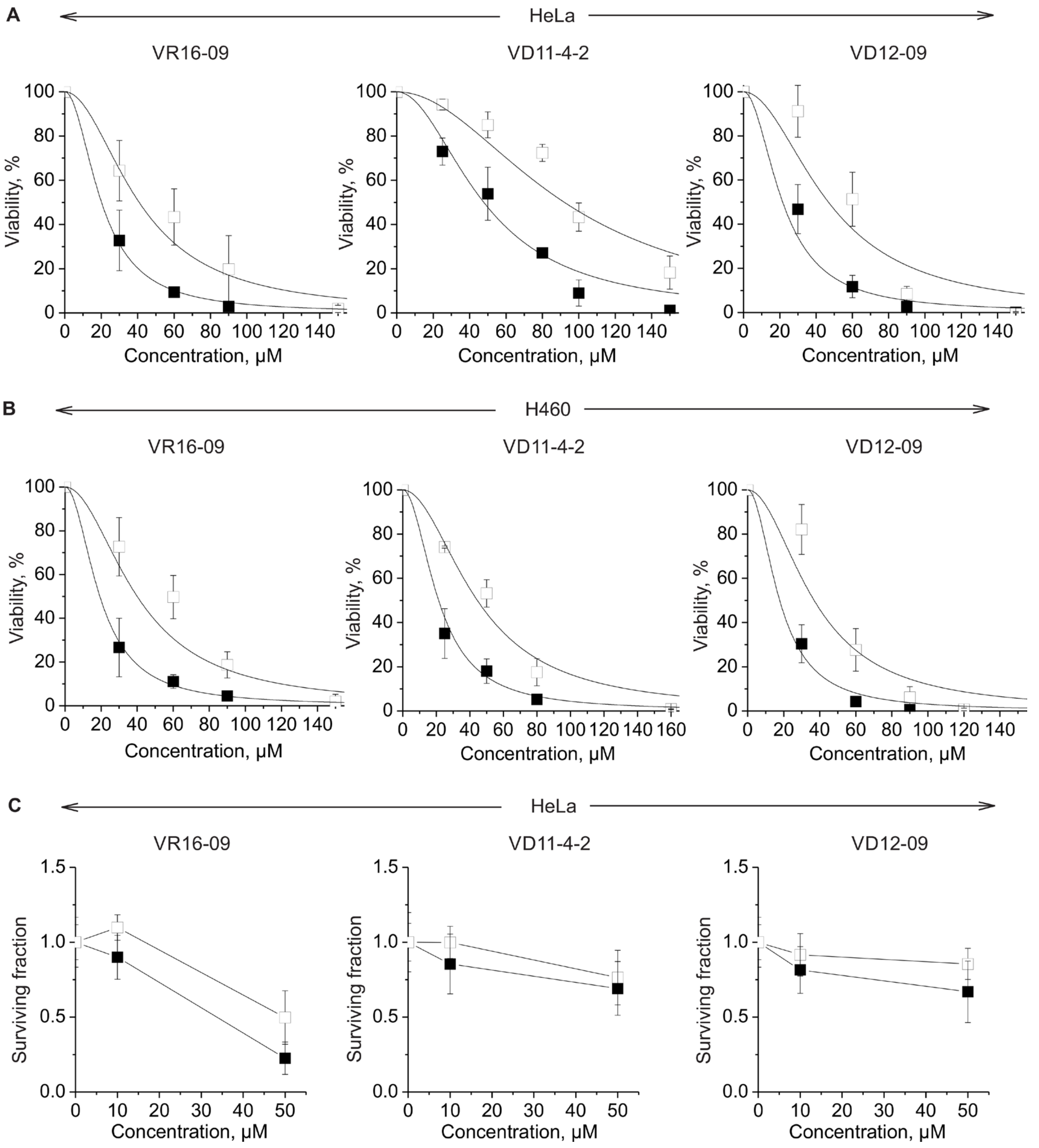

Supplementary Figure 6: Cytotoxicity profiles of compounds. (**A**) Dose-response curves representing the cytotoxicity of VR16-09, VD11-4-2, and VD12-09 in HeLa cells after 72 h exposure in normoxia (21% O_2; ■) or hypoxia (0.2% O_2; □). (**B**) Dose-response curves showing the cytotoxicity of compounds for H460 cells after 72 h exposure in normoxia or hypoxia. (C) Clonogenic survival of monolayer HeLa cells after the exposure to compounds for 72 h under normoxia and hypoxia. Surviving fraction was normalized to vehicle control. Average ± SD of 3 independent repeats is shown.

Supplementary Table 1: X-ray crystallographic data processing, refinement, and validation statistics for CA IX in complex with VR16-09, VR16-10, VD11-4-2, VD12-09, and without inhibitor

Structure	CA IX-VR16-09	CA IX-VR16-10	CA IX-VD11-4-2	CA IX-VD12-09	CA IX-apo
Space group	H3	H3	H3	H3	H3
Cell dimensions					
a = b (Å)	152.4	152.4	152.0	152.3	152.7
c (Å)	171.8	171.8	172.2	171.5	170.7
Resolution (Å)	76.2–2.47	76.2–1.75	25–1.95	26–1.91	31.7–1.87
Highest resolution shell (Å)	2.47–2.60	1.75–1.84	1.95–2.06	1.91–2.01	1.87–1.97
Number of reflections	53252	146413	107506	112458	121775
Number of reflections in test set	3768	7170	5404	5544	6280
Completeness (%)	99.8 (99.6*)	97.6 (95.5*)	99.4 (98.7*)	97.1 (98.3*)	99.3 (99.1*)
R_{merge}	0.17 (0.81*)	0.06 (0.50*)	0.07 (0.50*)	0.08 (0.54*)	0.05 (0.48*)
$\langle I/\sigma I \rangle$	7.7 (2.0*)	9.3 (2.0*)	8.7 (2.0*)	8.2 (2.0*)	9.9 (2.0*)
Average multiplicity	4.9 (4.8*)	3.0 (2.9*)	3.0 (2.7*)	2.7 (2.7*)	2.7 (2.7*)
R-factor	0.17 (0.29*)	0.17 (0.28*)	0.18 (0.34*)	0.17 (0.28*)	0.17 (0.27*)
R_{free}	0.21 (0.31*)	0.19 (0.27*)	0.22 (0.33*)	0.20 (0.29*)	0.20 (0.28*)
Average B factor (Å^2)	39.8	32.3	38.7	29.6	34.5
Average B factor for inhibitor (Å^2)	50.6	37.3	47.4	35.9	-
$\langle B \rangle$ from Wilson plot (Å^2)	35.3	23.1	24.8	23.1	26.8
Number of protein atoms	7397	7402	7736	7451	7714
Number of inhibitor atoms	152	136	112	104	-
Number of solvent molecules	422	880	736	792	763
r.m.s. deviations from ideal values					
Bond lengths (Å)	0.01	0.01	0.01	0.01	0.01
Bond angles (°)	1.45	1.40	1.51	1.48	1.38
Outliers in Ramachandran plot (%)	0.1	0.00	0.31	0.00	0.1
PDB code	6G98	6G9U	6FE1	6FE0	6FE2

*Values in parenthesis are for the high resolution shell.

Supplementary Table 2: EC_{50} values for VR16-09, VR16-10, VD11-4-2, and VD12-09 evaluated by alamarBlue® cell viability assay

	EC_{50}, µM											
	VR16-09			VR16-10			VD11-4-2			VD12-09		
**[O_2]**	21%	0.2%	CR	21%	0.2%	CR	21%	0.2%	CR	21%	0.2%	CR
**HeLa**	20.2 ± 4.3	40.8 ± 9.6	0.46 ± 0.06	93.3 ± 5.8	>150	>0.62	47.8 ± 9.6	92.2 ± 8.3	0.52 ± 0.08	21.6 ± 4.1	46.9 ± 7.0	0.44 ± 0.10
**H460**	19.3 ± 3.6	40.0 ± 9.6	0.49 ± 0.04	83.3 ± 15.3	>150	>0.55	21.4 ± 3.1	44.6 ± 6.4	0.48 ± 0.05	17.8 ± 2.2	37.0 ± 7.6	0.46 ± 0.09
**A549**	17.0 ± 1.0	76.7 ± 2.9	0.22 ± 0.02	ND	ND	ND	43.8 ± 2.5	105 ± 6	0.42 ± 0.04	33.8 ± 2.5	98.8 ± 8.5	0.34 ± 0.04
**MDA-MB-231**	74.2 ± 3.8	84.2 ± 4.9	0.88 ± 0.03	ND	ND	ND	52.8 ± 7.1	64.3 ± 11.5	0.83 ± 0.08	55.4 ± 6.6	67.6 ± 7.1	0.82 ± 0.09
**AsPC-1**	153 ± 23	160 ± 17	0.96 ± 0.12	ND	ND	ND	165 ± 6	145 ± 6	1.14 ± 0.09	100 ± 20	83.3 ± 5.8	1.19 ± 0.17

Cytotoxic ratios (CR) of selectivity to hypoxia are indicated. The HeLa, H460, A549, and MDA-MB-231 cells were exposed to the compounds under normoxia (21% O_2) or hypoxia (0.2% O_2) for 72 h, while AsPC-1 cells – for 48 h. Results of at least 3 independent repeats are shown (mean ± SD). ND – not determined.
